# Development of a High Oleic Cardoon Cell Culture Platform by *SAD* Overexpression and RNAi-Mediated *FAD2.2* Silencing

**DOI:** 10.3389/fpls.2022.913374

**Published:** 2022-06-20

**Authors:** Elisa Cappetta, Monica De Palma, Rosa D’Alessandro, Alessandra Aiello, Raffaele Romano, Giulia Graziani, Alberto Ritieni, Dario Paolo, Franca Locatelli, Francesca Sparvoli, Teresa Docimo, Marina Tucci

**Affiliations:** ^1^National Research Council, Institute of Bioscience and Bioresources, Portici, Italy; ^2^Department of Agricultural Sciences, University of Naples Federico II, Portici, Italy; ^3^Department of Pharmacy, University of Naples Federico II, Naples, Italy; ^4^National Research Council, Institute of Agricultural Biology and Biotechnology, Milan, Italy

**Keywords:** MUFA, cardoon calli, metabolic engineering, desaturases, oil crops, biorefinery

## Abstract

The development of effective tools for the sustainable supply of phyto-ingredients and natural substances with reduced environmental footprints can help mitigate the dramatic scenario of climate change. Plant cell cultures-based biorefineries can be a technological advancement to face this challenge and offer a potentially unlimited availability of natural substances, in a standardized composition and devoid of the seasonal variability of cultivated plants. Monounsaturated (MUFA) fatty acids are attracting considerable attention as supplements for biodegradable plastics, bio-additives for the cosmetic industry, and bio-lubricants. Cardoon (*Cynara cardunculus* L. var. *altilis*) callus cultures accumulate fatty acids and polyphenols and are therefore suitable for large-scale production of biochemicals and valuable compounds, as well as biofuel precursors. With the aim of boosting their potential uses, we designed a biotechnological approach to increase oleic acid content through *Agrobacterium tumefaciens*-mediated metabolic engineering. Bioinformatic data mining in the *C. cardunculus* transcriptome allowed the selection and molecular characterization of *SAD* (stearic acid desaturase) and *FAD2.2* (fatty acid desaturase) genes, coding for key enzymes in oleic and linoleic acid formation, as targets for metabolic engineering. A total of 22 and 27 fast-growing independent *CcSAD* overexpressing (OE) and *CcFAD2.2* RNAi knocked out (KO) transgenic lines were obtained. Further characterization of five independent transgenic lines for each construct demonstrated that, successfully, *SAD* overexpression increased linoleic acid content, e.g., to 42.5%, of the relative fatty acid content, in the *CcSAD*OE6 line compared with 30.4% in the wild type (WT), whereas *FAD2.2* silencing reduced linoleic acid in favor of the accumulation of its precursor, oleic acid, e.g., to almost 57% of the relative fatty acid content in the *CcFAD2.2*KO2 line with respect to 17.7% in the WT. Moreover, *CcSAD*OE6 and *CcFAD2.2*KO2 were also characterized by a significant increase in total polyphenolic content up to about 4.7 and 4.1 mg/g DW as compared with 2.7 mg/g DW in the WT, mainly due to the accumulation of dicaffeoyl quinic and feruloyl quinic acids. These results pose the basis for the effective creation of an engineered cardoon cells-based biorefinery accumulating high levels of valuable compounds from primary and specialized metabolism to meet the industrial demand for renewable and sustainable sources of innovative bioproducts.

## Introduction

Fossil materials are not renewable, and the plethora of derived industrial products that are in nearly everything we use are unsustainable for the environment and human lives. Therefore, there is a need to develop alternative bio-based materials from renewable sources (Isikgor and Becer, 2015). Vegetable oils, besides their paramount importance for human and animal nutrition, represent a valuable renewable resource for chemical feedstocks, since fatty acids, oils, and waxes possess chemical structures suited for the replacement of structural analogous petroleum-based molecules (Zhu et al., 2016). Oils enriched in unsaturated fatty acids (UFAs) (i.e., oleic acid) have significant industrial potential because of their stable chemical properties and high combustion value, allowing their direct use as bio-lubricants or biodiesel. Alongside, they are valuable as backbones for the formation of bio-based polymers (Rosenboom et al., 2022). Plant-based oil production for chemical feedstocks is strictly dependent on land availability. The development of biotechnological strategies oriented toward green chemistry, according to the principles of Circular Economy, is considered an alternative to boost plant oil production without compromising the food supply, in the context of the “land for food or fuel” debate, notwithstanding consumer concerns about the use of genetically modified (GM) organisms, as well as commercial and regulatory issues related to their exploitation, which mainly consider food products. Biotechnological strategies to manipulate UFAs accumulation include overexpression of enzymes of the fatty acid (FA) biosynthesis pathway, perturbation of the regulation of related biosynthetic pathways, or block of competing pathways (Bhati et al., 2021). The success of these approaches relies on a thorough mining of the key genes that determine oleic acid levels for the selection of the appropriate targets.

The UFAs are major components of the cell membrane, usually contain one or more double/triple carbon-carbon bonds in their acyl chains at specific locations, and are typically biosynthesized by a unique dehydrogenation reaction called desaturation. This process occurs in the plastid and the endoplasmic reticulum (ER) through two spatially independent pathways (Hildebrand, 2011) and is catalyzed by soluble and membrane-bound fatty acid desaturases (Murphy, 1999). In the fatty acid biosynthesis pathway, stearoyl-acyl carrier protein desaturases (SADs) catalyze the first desaturation step with the conversion of stearic acid (C18: 0) to oleic acid (C18: 1) by adding a cis-double bond between C9 and C10 of the carbon chain. The monounsaturated FA oleic acid can be further desaturated to polyunsaturated FAs. Thus, SADs play an important role in determining seed oil content and composition because of their significant effects on the ratio of saturated to unsaturated fatty acids (Smith et al., 2013). Given the functional importance of FA saturation in plant development and industrial application, homologous *SAD* genes from many plant species have been identified and characterized (Knutzon et al., 1992; Gibson, 1993; Tong et al., 2006; Schlüter et al., 2011; Liu et al., 2019; Li et al., 2022). However, even though all plants require the constitutive expression of *SAD* genes for oleic acid biosynthesis, the exact phylogenetic origin of these archetype acyl-ACP desaturases still remains unclear (Sperling et al., 2003) mainly due to the potential redundancy of multiple *SAD* isoforms (Cai, 2020). Further desaturation of oleic acid to linoleic acid (18: 2) is catalyzed by Δ12-desaturase/ω6-fatty acid desaturase, FAD2, and FAD6 in the plastid, while linoleic acid desaturation to γ-linolenic acid (C18: 3, n3) is catalyzed by Δ15-desaturase/ω3-fatty acid desaturase, FAD3, FAD7, and FAD8 in the plastid (Zhang et al., 2012; Bhunia et al., 2016). Although *FAD*s are all together responsible for membrane lipid alteration and adjustment, FAD2 plays a key role in catalyzing the desaturation of oleic acid to produce linoleic acid (He et al., 2020). *FAD2* gene was first reported in *Arabidopsis* with a single constitutively expressed copy (Okuley et al., 1994). Further studies have identified more than one *FAD2* gene in several crops (Kinney et al., 2002; Hernández et al., 2005; Rolletschek et al., 2007; Kargiotidou et al., 2008), which have been classified into three types based on their site and pattern of expression. *FAD2-1* is a seed-specific desaturase (Liu et al., 2001), *FAD2-2* is constitutively expressed in both developing seeds and vegetative tissues (Hernández et al., 2009; Dar et al., 2017), while *FAD6* shows a higher transcript level in leaves (Chi et al., 2011). Differences in tissue expression levels assigned the major role for the conversion of oleic to linoleic acid in the ER to *FAD2-2*, while the *FAD6* gene could be implicated in the oleic acid desaturation of leaf plastidial lipids (Falcone et al., 1994; Ohlrogge and Browse, 1995). Oilseed crops contain saturated acids (C16: 0 and C18: 0), oleic acid (C18: 1), linoleic acid (C18: 2), and α-linolenic acid (C18: 3) as the main FAs in their seed oil. Non-food seed oil species tobacco (*Nicotiana tabacum*) and camelina (*Camelina sativa*) showed the presence of stearic acid (∼3%) in both species and oleic acid of 14.5 and 16.7%, respectively, as main fatty acids (Giannelos et al., 2002; Rodríguez-Rodríguez et al., 2013). Since these species can provide oil for industrial raw materials, high oil quality is desirable by improving complex traits such as seed oil content and fatty acid composition through conventional breeding or molecular biology to obtain germplasm with elevated MUFAs, e.g., oleic acid, and reduced PUFAs (Heddleson and Kodali, 2022). In the last years, genetic manipulation of desaturase genes involved in the key step of fatty acid desaturation has addressed the modification of the fatty acid flux, especially in favor of MUFAs accumulation, with varying degrees of success (Jadhav et al., 2005; Vanhercke et al., 2013; Klinkenberg et al., 2014; Yuan and Li, 2020). For example, overexpression of the *SAD* gene triggered the synthesis of relevant PUFAs in *Arabidopsis*, potato, and camelina (De Palma et al., 2008; Klinkenberg et al., 2014; Rodríguez-Rodríguez et al., 2021). Alternatively, targeted downregulation of *FAD2* genes using RNA interference in rapeseed and soybean boosted the seed oil oleic acid content up to 85% (Peng et al., 2010; Zhang et al., 2014). Recently, advanced seed lipid engineering programs by CRISPR/Cas9 have been successfully adopted to produce high oleic seed oil by knockout of *FAD2* genes in a variety of crop species (Okuzaki et al., 2018; Do et al., 2019; Tian et al., 2020).

Sustainable biorefineries based on oil crops cell cultures could be a valuable alternative to field cultivation, providing large-scale production of fatty acids along with other bioactive molecules to be used as bioplastics and biofuel precursors, as well as biochemicals and valuable compounds, without causing detrimental land-use changes and devoid of the seasonal variability in the composition of cultivated plants. As regards, cell cultures from globe artichoke have been already explored as a valuable and innovative source of natural antioxidants (Meneghini et al., 2016). Among oil crop species, cultivated cardoon is considered one of the most promising biomass crops in the Mediterranean, which is able to grow in harsh habitat conditions such as high temperature, elevated salinity, and arid summer (Benlloch-González et al., 2005). One of the most interesting features of cardoon biomass is its oil content (mainly from seeds). Cardoon oils, thanks to their high unsaturated to saturated fatty acids ratio, high levels of oleic (from ∼17 to 22%) and linoleic acids (from ∼20 to 61%), and stability to oxidation (Maccarone et al., 1999; Angelova et al., 2019; Graziani et al., 2020), can be used in the nutraceutical field, lowering serum cholesterol levels (Lavermicocca et al., 2010) and in biorefinery, e.g., for the production of biodegradable plastics (Wellenreuther and Wolf, 2020). However, as a crop-derived commodity, cardoon biomass suffers from seasonal availability and variable yield quality and quantity. For these reasons, the development of cardoon cell cultures for biorefinery applications could be a valuable alternative strategy. A significant step toward the industrial use of cardoon cell cultures was recently obtained by Paolo et al. (2021), who obtained AtMYB4 overexpressing callus lines characterized by the faster growth rate, reduction in lignin content, and improved accessibility of the biomass to enzymatic degradation.

In our study, we designed strategies to direct metabolic fluxes toward a higher unsaturated to saturated fatty acids ratio in cardoon cells. Increased oleic acid content was addressed through metabolic engineering of fatty acid biosynthesis. Specific fatty acid biosynthesis genes, *SAD* and *FAD2.2*, were selected and used to transform cardoon leaf-derived calli through *A. tumefaciens*. *SAD*-overexpressing and *FAD*-silenced transgenic calli lines were molecularly and metabolically characterized to verify the metabolic flux toward the higher accumulation of oleic acid, confirming increased accumulation of unsaturated fatty acids and ameliorated content in bioactive phenylpropanoids.

## Materials and Methods

### Gene Identification, Phylogenetic Analysis, and Protein Prediction

#### Sequence Retrieval and Chromosomal Location of the *SAD* and *FAD* Gene Families

BLASTp (Altschul et al., 1990) was used to search homologs of the *Arabidopsis thaliana* AtSAD (AT1G43800) and AtFAD2 (AT3G12120, AT4G30950.1) predicted amino acid sequences in the *C. cardunculus* genome [Annotation (Acquadro et al., 2020) on v2.0 assembly] using proteome and genome files downloaded from the Globe Artichoke Genome Database^1^.

The scaffold for all seventeen chromosomes and the General Feature Format (GFF) file (v2.0 – Genomic features.gff) were also retrieved from the Globe Artichoke Genome Database and used for mapping *SAD* and *FAD2* genes. These genes were graphically portrayed on the chromosomes using the “PhenoGram” tool of Ritchie Lab^2^ (Wolfe et al., 2013).

We renamed all genes based on the chromosome position, as shown in Supplementary Figure 1. Other features of the identified *Cc*SAD and *Cc*FAD2, such as isoelectric points (pIs), molecular weights (MWs), and average hydropathicity (GRAVY), were calculated using ExPASy (Gasteiger et al., 1999; Supplementary Table 1).

#### Phylogenetic Tree Construction, Domain Architecture, and Motif Analysis of Desaturase Genes

All SAD and FAD2 protein sequences identified in *C. cardunculus* and protein sequences from several species, including many oilseed crops (Supplementary Table 2), were aligned using the ClustalW algorithm with default parameters^3^ (Thompson et al., 1994). A phylogenetic tree was carried out from the multiple sequence alignments using MEGA X (Kumar et al., 2018) and was inferred with neighbor-joining (NJ) method. The bootstrap consensus tree was built using 1,000 replicates. Branches corresponding to partitions reproduced in less than 30% of bootstrap replicates were collapsed. *Volvox carteri* sequences were used as an outgroup in the species tree.

Protein conserved domains of *C. cardunculus* desaturases genes were analyzed using the Pfam protein family database (Pfam 24.0) (Punta et al., 2012). Predictions of motifs were generated using the Multiple Em for Motif Elicitation (MEME) program^4^ (Bailey et al., 2015), with the maximum number of motifs set to 6 and default values for other parameters.

### Plant Material and Growth Conditions

Friable callus was induced from cardoon leaves of the cv “Spagnolo” as described (Paolo et al., 2021) and maintained in the dark at 25°C on Gamborg B5 (GB5) growth medium including vitamins (Duchefa Biochemie, Haarlem, Netherlands), supplied with 1 mg/L 2,4-dichlorophenoxyacetic acid (2,4-D), 1 mg/L adenine, 0.1 mg/L kinetin, 3% (w/v) sucrose, and 8% (w/v) agar, adjusted to pH 5.8.

### RNA Extraction and qRT PCR Analysis of Gene Expression

Total RNA was extracted from 100 mg of friable callus using 1 mL of TRIzol extraction buffer (Thermo Fisher Scientific, Wilmington, DE, United States) according to the manufacturer’s protocol. RNA concentration was measured with a NanoDrop ND-1000 spectrophotometer (Thermo Fisher Scientific, Wilmington, DE, United States). Reverse transcribed DNase-treated total RNA (1 μg) was obtained using SuperScript II Reverse Transcriptase (Life Technologies, Carlsbad, CA, United States). RT-qPCR was performed in an ABI7900 HT (Life Technologies, CA, United States). Each PCR consisted of 2 μl of 1: 25 diluted cDNA, 10 μl of 2X PowerUp™ SYBR™ Green Master Mix (Applied Biosystems, CA, United States), and 0.4 μM of each gene-specific primer, listed in Supplementary Table 3 in a total volume of 20 μl. The thermal cycling conditions were 50°C for 2 min (one step), one cycle at 95°C for 10 min, followed by 40 cycles of two steps at 95°C for 15 s and 60°C for 1 min. PCR product melting curves (60–95°C) were analyzed for the presence of a single peak. Three technical repetitions were tested for each sample. To normalize gene expression values, the geometric mean of three internal controls as relevant reference genes was used (Supplementary Table 3). Expression was calculated by the 2^–ΔΔ*CT*^ method (Pfaffl, 2001, 2004). For calli *vs*. leaf tissue *CcSAD* and *CcFAD* transcript levels, relative expressions were calculated using leaf tissue as calibrator. Expression levels of *CcSAD* and *CcFAD* transcripts for transgenic calli were compared with the untransformed line (WT) used as calibrator.

### Fatty Acids Profile

The fatty acids profile was determined after extracting the fat and subsequent trans-esterification to obtain the methyl esters of the fatty acids (according to IUPAC standard method no. 2.301). The extraction was performed using the Folch method (Folch et al., 1957) with some modifications. Specifically, approximately 100 mg of lyophilized *C. cardunculus* calli were placed in a 15 ml centrifuge tube and added with 3 ml of a chloroform-methanol mixture (2: 1 v/v) and 0.1% of butylated hydroxytoluene (BHT). After shaking with a vortex for 6 min, 1 ml of a sodium chloride saturated solution was added to the mixture. After centrifuging at 8,000 × *g* for 10 min, the subnatant was collected in a flask. The extraction protocol was repeated three times. The flask content was dried in a rotary evaporator at 36°C. The extracted lipid was then dissolved in hexane to prepare a 1% solution. This solution was subjected to transesterification with a 2 M KOH solution in methanol and gas chromatographic analysis. Details on methodologies, instrumentation used, and peak identification were described in Romano et al. (2021), with some differences regarding the oven temperatures (70°C for 1 min, 20°C for 1 min, ramp to 140°C for 5 min, and then 7°C for 1 min, ramp to 240°C for 10 min). The results were expressed as % w/w.

### Plasmid Expression Constructs for *CcSAD* Overexpression and *CcFAD2.2* RNAi Strategies

The coding sequence of the genes of *CcSAD* (1,149 bp) was amplified from *C. cardunculus* young leaves cDNA by PCR using a Phusion HF DNA Polymerase (Thermo Fisher Scientific, Waltham, MA, United States) and specific primers (Supplementary Table 3) characterized by the presence of the short sequence CACC at 5’ for the cloning in pENTR/kit D-TOPO^®^ (Invitrogen) to generate an Entry-Clone. Subsequently, the coding sequence was subcloned through the LR reaction (The Gateway^®^ LR Clonase™ enzyme mix kit, Invitrogen) in the gateway Destination vector PGWB411, driven by the constitutive strong viral 35SCaMV promoter, and containing the kanamycin resistance selectable marker (Schardl et al., 1987) through site-specific recombination (Xu and Qingshun, 2008). The resulting binary vectors were shuttled into the *A. tumefaciens* (strains GV3101) by the standard freeze-thaw method (Hofgen and Willmitzer, 1988). The cloning of the partial sequence (768 bp) of the *CcFAD2.2* gene into pHELLSGATE12 RNAi binary vector (characterized by the presence of the constitutive strong viral 35SCaMV and kanamycin resistance) was performed with the same procedure (gateway technology-based) described above for the cloning of *CcSAD*.

### Agrobacterium-Mediated Cardoon Calli Transformation and Identification of Transformants

The *A. tumefaciens* strain GV3101, kindly provided by Dr. M.S Grillo of CNR-IBBR, was grown on yeast extract peptone (YEP) solid medium containing 30 mg/L gentamicin and 25 mg/L rifampicin at 28°C and used for all genetic transformation experiments. Overnight culture of GV3101 carrying the pGWB411: *CcSAD* or pHELLSGATE12: *CcFAD2.2* construct and their respective empty vectors were inoculated into 30 ml of YEP containing 100 mg/L kanamycin, 30 mg/L gentamicin, and 25 mg/L rifampicin and allowed to grow at 28°C. *A. tumefaciens* cells were collected when the OD_600_ value reached approximately 0.60–0.90 by centrifugation at 6,000 × *g* for 10 min at 4°C and resuspended at OD_600_ 0.8 value with liquid GB5 medium. Ten days subcultured cardoon calli were agro-infected with GV3101 suspension by co-incubation in the presence of 100 μM acetosyringone (Sigma Aldrich, MO, United States), shaking on a rotary shaker at 150 rpm for 1 h at 28°C. Then, calli were placed on sterile filter paper to remove the liquid excess and transferred onto GB5 solid medium at 28°C. Two days agro-infected calli were spread on a selection medium containing cefotaxime (250 mg/L) and kanamycin (100 mg/L) and subcultured approximately every 21 days to a fresh selective medium. For each construct, three distinct transformation experiments were carried out.

Antibiotic-resistant calli were individually maintained on GB5 selective solid medium and analyzed by PCR to confirm the presence of the transgene. Genomic DNA was obtained from 100 mg of friable callus of untransformed (wild type, WT) and putative transgenic calli using the DNeasy Plant Mini Kit (Qiagen, Germany) according to the manufacturer’s protocol. Each PCR was prepared in a total volume of 25 μl using Phusion Hot Start II DNA Polymerase (Thermo Fisher Scientific, Wilmington, DE, United States) and specific primers to either of the transgenes and kanamycin resistance gene (Neomycin phosphotransferase II, NPTII) (Supplementary Table 3). PCR amplification was performed in a 2720 Thermal Cycler (Applied Biosystems, CA, United States), with the following cycle: 1 cycle at 98°C for 30 s, followed by 30 cycles of three steps at 98°C for 10 s, 58°C for 20 s, 72°C for 30 s, and final extension at 72°C for 10 min. The absence of any residual contamination by *Agrobacterium* was confirmed by growing all the transgenic lines on YEP selective liquid medium at 28°C on a rotary shaker at 150 rpm for 48 h.

### Polyphenols Extraction and UHPLC-HRMS Analysis

Freeze-dried material was extracted by ultrasound-assisted extraction as previously reported (Graziani et al., 2020). In brief, 3 g of freeze-dried samples were extracted in 30 ml of ethanol/water (50:50, v/v) and then sonicated for 30 min at room temperature. The extracts were centrifuged at 3,000 × *g* for 10 min at 4°C; the supernatant was filtered through a 0.22 μm nylon membranes syringe filter (Phenomenex, Castel Maggiore, BO, Italy) and used for UHPLC-HRMS analysis. Quali-quantitative analysis of polyphenols extracted from cardoon cell cultures was performed using a UHPLC system (Thermo Fisher Scientific, Waltham, MA, United States) equipped with a degassing system, a quaternary UHPLC pump, and an autosampler device (Dionex Ultimate 3000). Chromatographic separation of polyphenols was performed at 25°C, using a Luna Omega 1.6 μm PS column (50 mm × 2.1 mm, Phenomenex, Torrance, CA, United States). Polyphenols were eluted with two mobile phases consisting of water with 0.1% formic acid v/v (Phase A) and acetonitrile with 0.1% formic acid v/v (Phase B). A gradient mobile phase was used as follows: initial 0% B for 1 min, increased to 95% B in 1 min. The gradient was held for 0.5 min at 95% B and linearly decreased to 75% B in 2.5 min and decreased again to 60% B in 1 min. Afterward, the gradient switched back to 0% B in 0.5 min and was held for 2.5 min for column re-equilibration. The flow rate was 0.4 ml/min, and the injection volume was 5 μl.

The UHPLC system was coupled to a Q-Exactive Orbitrap Mass Spectrometer (UHPLC, Thermo Fischer Scientific, Waltham, MA, United States) equipped with an electrospray (ESI) source operating in negative mode (Thermo Fisher Scientific, Bremen, Germany). The acquisitions were conducted by setting full MS/all ion fragmentation (AIF) mode. Full MS experiments were carried out with the settings: microscans, 1; automatic gain control (AGC) target, 1e6; maximum injection time, 200 ms; mass resolution, 35,000 full widths at half maximum (FWHM) at *m/z* 200, whereas AIF scan conditions were as follows: microscans, 1; AGC target, 1e5; maximum injection time, 200 ms; mass resolution, 17,500 FWHM at *m/z* 200, HCD energy, at 10, 20, and 45. In both cases, the instrument was set to spray voltage 3.5 kV, capillary temperature 275°C, sheath gas 45 (arbitrary units), auxiliary gas 10 (arbitrary units), *m/z* range 80–1,200, and data acquisition in profile mode. The accuracy and calibration of the Q Exactive Orbitrap LC-MS/MS were checked on a daily basis before the analysis of samples using a reference standard mixture. For the calibration of the mass spectrometer, the PierceTM LTQ Velos Electrospray Ionization (ESI) positive and negative ion calibration solutions (Thermo Fisher Scientific) were used. In particular, ESI positive ion calibration solution consisted of caffeine (20 μg/ml), MRFA (1 μg/ml), and Ultramark 1621 (0.001%) in an aqueous solution of acetonitrile (50%), methanol (25%), and acetic acid (1%), while ESI negative calibration solution consisted of sodium dodecyl sulfate (2.9 μg/ml), sodium taurocholate (5.4 μg/ml), and Ultramark 1621 (0.001%) in an aqueous solution of acetonitrile (50%), methanol (25%), and acetic acid (1%). The mass tolerance window was set to 5 ppm in both full scan MS and AIF modes. Data analysis and processing were performed using the Xcalibur software v. 3.1.66 10 (Xcalibur, Thermo Fisher Scientific, Waltham, MA, United States). Metabolites quantitation was calculated using calibration curves from authentical standards when available, otherwise based on calibration curves of standard compounds belonging to the same chemical group and with a similar response. All values were expressed in μg/g DW.

### Calli Fresh Weight and Relative Growth Rate Measurements

Biomass changes of untransformed calli (WT) and transgenic lines were determined by recording calli fresh weight from three biological replicates (*n* = 8–10 for each biological replication). Weight was recorded at the time of calli transfer (T0) on fresh GB5 medium and every 7 days until 28 days of culture (T0–T4). For growth rate measurements of WT and *CcSAD*OE6 and *CcFAD2.2*KO2 transformed lines, weights were measured on two biological replicates (*n* = 8–10 for each biological replication), and the mean callus relative growth rate (GR) was calculated by [(T final-T initial)/T initial] × 100% according to (Golkar et al., 2019).

### Statistical Analysis

One-way ANOVA and Tukey’s multiple-range test (*p* ≤ 0.05) were conducted on the data obtained from fatty acid determination using the XLSTAT software (Addinsoft, New York, NY, United States). Biochemical data, qRT-PCR data, and growth rate analysis for calli lines were subjected to ANOVA by the Sigma Plot software (SAS Institute, Cary, NC, United States). Means were compared using Tukey’s HSD test (*p* ≤ 0.05).

### Data Availability Statement

The original contributions presented in this study are included in the article/Supplementary Material, further inquiries can be directed to the corresponding author.

## Results

### Genome-Wide Identification, Chromosomal Location, and Sequence Features of the *SAD* and *FAD2* Members in *Cynara cardunculus*

**FIGURE 1:**
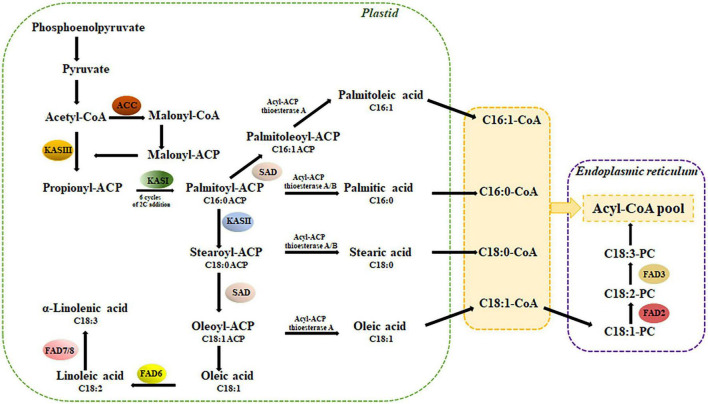
Simplified biosynthetic pathway of fatty acids in plants (based on Dar et al., 2017): ACC, acetyl-CoA carboxylase; KAS, ketoacyl-ACP synthase; SAD, stearoyl-ACP desaturase; FAD, fatty acid desaturase; PC, phosphatidylcholine.

The key enzymes in the biosynthesis of valuable oleic and linoleic unsaturated fatty acids are encoded by *SAD* and *FAD2* genes (Figure 1). To select *SAD* and *FAD* candidate genes, first we investigated the abundance and evolutionary pattern of *CcSAD* and *CcFAD2* genes in the *C. cardunculus* genome. For this purpose, *A. thaliana* SAD and FAD2 proteins were used as queries to blast the *C. cardunculus* genome database. A total of four and sixteen genes were identified as *CcSAD* and *CcFAD2*, respectively. These latter *CcFAD2* genes coded for either plastidial (FAD6) or microsomal (FAD2) fatty acid desaturases enzymes.

The chromosomal positioning of *CcSAD* and *CcFAD2* genes and initiation sites were determined using the PhenGram online server (Supplementary Figure 1). We renamed all genes based on the chromosome position. Among the 17 *C. cardunculus* chromosomes, two out of four *CcSAD* genes were located on chromosome 2 (*CcSAD1* and *CcSAD2*), while Chr3 and Chr10 contained *CcSAD3* and *CcSAD4*, respectively. On the contrary, the sixteen *CcFAD2* genes were found located on 5 chromosomes (Chr 2, Chr5, Chr8, Chr13, and Chr15).

The number of *CcFAD2* mapped on chromosomes 2, 5, 8, 13, and 15 was 4,1, 1, 6, and 4, respectively. Interestingly, part of these *FAD2.2* genes was organized in clusters, and three gene clusters were found on Chr 2, 13, and 15; each cluster harbored 4, 5, and 4 *CcFAD2* genes (Supplementary Figure 1).

The four *CcSAD* genes encoded proteins ranging from 382 aa (*Cc*SAD1 and *Cc*SAD2) to 396 aa (*Cc*SAD3 and *Cc*SAD4). The length of the protein sequences encoded by the *CcFAD* genes ranges from 374 amino acids of *Cc*FAD2.1.7 to 1,611 amino acids of CcFAD2.1.8. The predicted theoretical isoelectric points (pIs) ranged from 5.81 to 6.71 for *Cc*SAD proteins and from 6.26 to 9.41 for *Cc*FAD2, the molecular weights (MWs) of the *Cc*SAD proteins ranged from 43.4 to 44.99 kDa and from 43.67 to 187.39 for CcFAD2, and the average hydrophilicity ranged from −0.425 to −0.281 for *Cc*SADs and −0.137 to 0.110 for *Cc*FAD2 proteins (Supplementary Table 1).

### Phylogenetic Analysis and Conserved Motifs of *Cc*SAD and *Cc*FAD2 Proteins

**FIGURE 2:**
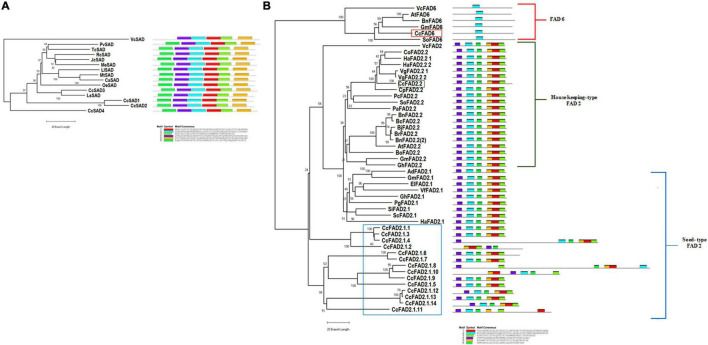
Phylogenetic tree (on the left) and motif composition (on the right) of *SAD* and *FAD* candidate genes from *Cynara cardunculus* and other 31 representative species (Species and Accession numbers are reported in Supplementary Table 2). The amino acid sequences of all the selected proteins were aligned using the ClustalW program and subjected to phylogenetic analysis by the neighbor-joining method using the MEGAX software. Motif composition of the *Cc*FAD and *Cc*SAD proteins was obtained by the MEME program. The gray line represents the length of the proteins, and the rectangles in different colors represent the different motifs from 1 to 6. **(A)** Phylogenetic tree and motif composition of *C. cardunculus* SADs plus other 10 plant species. Rooted phylogram was obtained by using the *Volvox carteri Vc*SAD sequence as the outgroup. **(B)** Phylogenetic tree and motif composition of *C. cardunculus* FADs plus other 21 plant species. The house-keeping *Cc*FAD2.2 protein is indicated by a green box, the seed-type *Cc*FAD2.1 proteins are indicated by a blue box, and the *Cc*FAD6 protein is indicated by a red box. Rooted phylogram was obtained by using the *Volvox carteri Vc*FAD2 and *Vc*FAD6 sequences as the outgroups for FAD2 and FAD6 analysis, respectively.

To investigate the evolutionary relationships of the *C. cardunculus* SAD and FAD2 proteins with other plant SADs and FADs, a total of 14 SAD and 48 FAD2 protein sequences from 31 representative species (including *C. cardunculus* and other oilseed crops) were used to generate phylogenetic trees, and to highlight evolutionary divergences respective to SAD and FAD proteins from the chlorophyta, *Volvox carteri* was used as outgroup (Figures 2A,B and Supplementary Table 2). In the SADs phylogenetic tree, the four *Cc*SAD isoforms clustered close to the SAD proteins from *Lactuca sativa* and *Olea europaea* (Figure 2A).

Domain architecture and motifs conservation of SAD protein sequences were investigated, and all the analyzed *Cc*SADs contained the same and unique fatty acid desaturase 2 (FA_desaturase_2) domain (pfam03405) (data not shown). In addition, all proteins showed the same motif structure and distribution, except for *Vc*SAD, which lacked motif-6. According to the FAD2 phylogenetic tree that included both plastidial (FAD6) and microsomal (FAD2) oleate desaturase enzymes, FAD proteins could be separated into three main groups: house-keeping FAD2, seed-type FAD2, and FAD6 (Figure 2B). FAD2 seed-type gene family, included most of the *Cc*FAD2 proteins (14), while the FAD6 and the house-keeping FAD2 enzymes in *C. cardunculus* are encoded each by a single gene, located on chromosomes 8 (*CcFAD6*) and 5 (*CcFAD2.2*). As a consequence of fatty acids biosynthesis conservation across eukaryotic organisms, FAD2 microsomal and FAD6 plastidial proteins clustered with *Vc*FAD2 and *Vc*FAD6 from algae, whereas, as expected, higher divergence was found for FAD2.1 seed type isoforms, which are present only in plants. To further analyze the conservation of these protein sequences and structures, the 16 *Cc*FAD2 proteins were subjected to a domain architecture analysis. The results showed that all the analyzed proteins contained one C-terminal FAD domain (FA_desaturase, pfam00487) except 5 *Cc*FAD2 proteins, all belonging to the seed-type *C. cardunculus* species, that contained from two to four FA_desaturase domains (data not shown). Using the MEME software (Figure 2B) to discover and search for conserved motifs, we found that all genes except *Cc*FAD2.1.2 contained the conserved motif-2. The remaining 5 motifs are present in all members of the FAD2 subfamilies, but not in the FAD6 family (Figure 2B).

### *Cynara cardunculus* Calli Accumulate Mono-Unsaturated Fatty Acids

**FIGURE 3:**
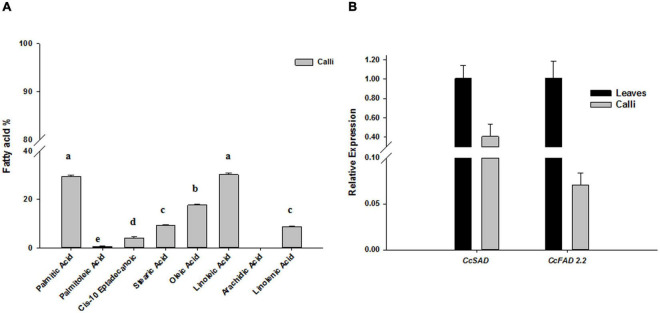
Fatty acid accumulation **(A)** and relative expression of *CcSAD* and *CcFAD2.2* biosynthetic genes **(B)** in *C. cardunculus* calli and leaves. The data are presented as mean ± SD (*n* = 3). Different letters indicate statistically significant differences (*p* < 0.05).

In our previous work, we showed that the cv “Spagnolo” accumulated a high content of palmitic acid 34%, oleic acid 15.5%, and linoleic acid 37.4% in leaves, with a ratio of monosaturated/unsaturated fatty acids (MUFA/PUFA) of about 0.37 (Graziani et al., 2020). To verify whether undifferentiated leaf-derived calli could have a comparable fatty acids profile and content, we performed a GC/MS analysis on calli after 28 days of subculture. We found that the most abundant fatty acids in cardoon calli were palmitic and linoleic acids, reaching about 29–30%, followed by oleic acid whose amount was about 17% (Figure 3A), overall resembling a profile similar to a leaf. The transcriptional activity of the major biosynthetic genes involved in monounsaturated fatty acids biosynthesis was also monitored in calli by qRT-PCR of *CcSAD* and *CcFAD2.2* isoforms, selected based on sequence similarity with other homologous proteins functionally characterized in other species, mostly oleaginous ones. *SAD* and *FAD2.2* genes were expressed also in calli, although their transcript levels were half and ten times reduced, respectively, compared with leaf tissues (Figure 3B).

### Increasing Mono-Unsaturated Fatty Acids in *Cynara cardunculus* Calli by *SAD* Overexpression and *FAD* RNAi Strategies

Based on previously described data, we considered leaf-derived calli of *C. cardunculus* as a suitable platform to build a biofactory for the optimized production of MUFA. To this end, we designed a biotechnological approach based on *A. tumefaciens*-mediated metabolic engineering to further increase their oleic acid levels by manipulating the expression of the *CcSAD* and *CcFAD2* desaturase genes selected following the bioinformatics analyses.

#### Generation of Transgenic Callus Lines Overexpressing the *CcSAD* Gene or RNAi-Silenced for the *CcFAD2.2* Gene

**FIGURE 4:**
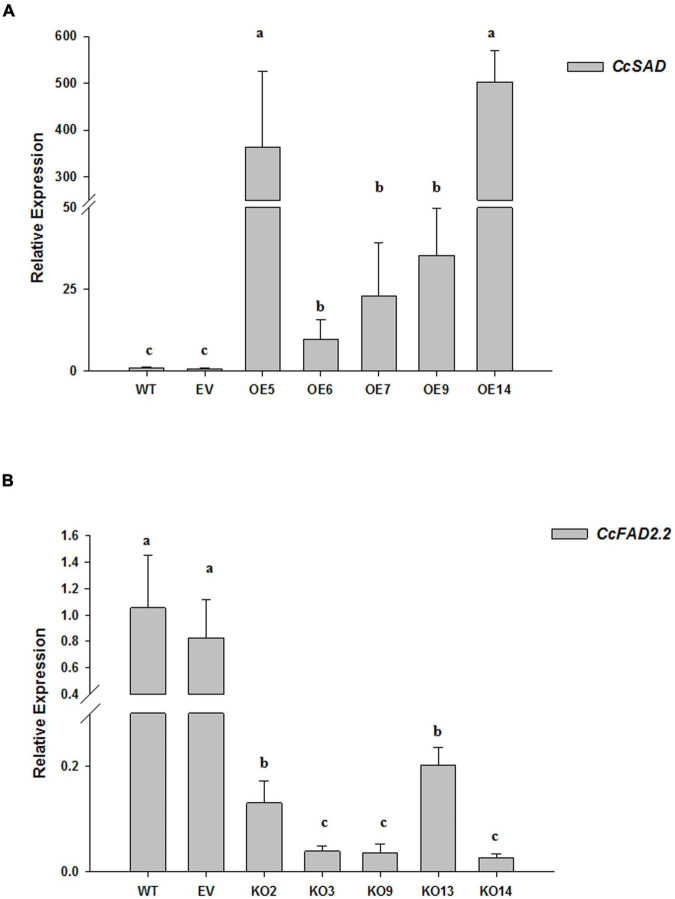
Relative expression of the Cc*SAD* **(A)** and *CcFAD2.2* **(B)** genes in the transgenic lines of *C. cardunculus* calli was performed by quantitative PCR (qPCR). Total RNA was extracted from non-agro-inoculated cardoon (WT) and the empty vector (EV) calli as controls, and from *CcSAD*OE lines (5, 6, 7, 9, 13, and 14) or *CcFAD2.2*KO lines (2, 3, 9, and 14). The data are presented as mean ± SD. Different letters indicate statistically significant differences (*p* < 0.05).

A stable *A. tumefaciens*-mediated transformation of cardoon leaf-derived calli was set up to obtain transformant lines overexpressing (OE) or knocked out (KO) for the coding sequence of the *C. cardunculus SAD* (*CcSAD1*) or *FAD2.2* (*CcFAD2.2*) genes, respectively. For each construct, 80 WT calli were co-cultivated with the GV3101 *A. tumefaciens* strain bearing either pGWB411: *CcSAD1* or pHELLSGATE12: *CcFAD2.2*. As a positive control of transformation, 30 WT calli were inoculated with *Agrobacterium* carrying the empty vectors pGWB411 or pHELLSGATE12. After 3 months from transformation under constant selection in the presence of kanamycin, 22 and 27 fast-growing independent callus lines for pGWB411: *CcSAD1* and pHELLSGATE12: *CcFAD2.2* were obtained, respectively. Ten lines for each construct were further analyzed by genomic PCR using primers specific to either of the transgenes or the kanamycin resistance gene, confirming that the exogenous expression cassettes were successfully integrated into *C. cardunculus* genomic DNA (Supplementary Figure 2). Based on visual observation of the growth and appearance of *C. cardunculus* calli, five independent Cc*SAD*OE and Cc*FAD2.2*KO transgenic lines for each construct were selected and used along with untransformed (WT) and empty vector-transformed (EV) calli for further studies. The expression of genes of interest for all evaluated transgenic calli was compared with WT calli used as calibrator. The expression of both genes of interest in EV calli and WT ones was not statistically different, hereafter we refer to both controls in our analyses. When evaluated by qRT-PCR, all the *CcSAD*OE lines actively expressed the transgene compared with WT and EV calli controls, with a large dynamic range varying from 20 to 500 fold change increase. The highest SAD expression was shown by *CcSAD*OE14 and *CcSAD*OE5 lines, whose fold change increased over 300 and 500, respectively. *SAD* expression in the other lines increased to lower levels, varying from about 10 to 35 times increased transcript levels (Figure 4A). All the *CcFAD2.2*KO lines showed a significant reduction of the *CcFAD2.2* translational activity when compared with the WT and EV calli controls. In particular, an expression reduction ranging from 10 to 50 times less was detected for *FAD2.2*KO2 and *FAD2.2*KO14, respectively (Figure 4B).

#### Fatty Acid Flux Modification in *CcSADOE* and *CcFAD2.2KO* Lines of *Cynara cardunculus*

The fatty acid (FA) profile, as shown in Supplementary Figures 3A,B, was represented by palmitic (C16: 0), palmitoleic (C16: 1), heptadecanoic (C17: 0), stearic (C18: 0), oleic (C18: 1), linoleic (C18: 2), linolenic (C18: 3), and arachidic acid (C20: 0). No significant qualitative and quantitative differences were detected between WT and EV transformed lines. On the contrary, *CcSAD*OE and *CcFAD2.2*KO calli significantly modified fatty acid profiles in all the five independent transformed lines as compared with WT and EV controls (Supplementary Figures 3A,B). In all *CcSAD*OE transgenic lines, the most abundant FAs were linoleic acid ranging from 52.5 to 42.5%, palmitic acid from 29.7 to 21.6%, oleic acid from 17.3 to 9.8%, and linolenic acid from 12.6 to 8.6%, whereas palmitoleic and arachidonic acids were undetected in most of the analyzed lines. Compared with controls, *CcSAD*OE calli showed an increase in the metabolic flux toward unsaturated fatty acids, with a significant depletion of palmitic, palmitoleic, and arachidonic acids (Supplementary Figure 3A). As regards, *CcSAD*OE6 showed a marked increase in linoleic acid without reducing the oleic acid content. Evaluation of *FAD2.2* silencing on fatty acid profile showed that, in all *CcFAD2.2*KO lines, the most present fatty acid was oleic acid ranging from 56.7% to 17.7%, followed by linoleic acid ranging from 43.7% to 18.6%. In parallel to the increase in oleic and linoleic acids, in *CcFAD2.2*KO lines, a significant depletion of saturated fatty acids was detected, compared with controls. Namely, palmitic and stearic acids were the most abundant fatty acids in control lines, whereas their amount was half and two times reduced in transformed lines (Supplementary Figure 3B). Among *CcFAD2.2* silenced lines, *CcFAD2.2*KO2 showed the highest accumulation of oleic acid, whereas in all the other silenced lines a minor but significant increase of oleic acid was accompanied also by a marked increase in linoleic acid (Supplementary Figure 3B). In accordance with the increase of oleic acid content by *CcSAD* overexpression and *CcFAD2.2* silencing, both strategies were successful, and this is particularly true for *CcSAD*OE6 and *CcFAD2.2*KO2 lines. *CcSAD*OE6 and *CcFAD2.2*KO2 transgenic lines showed a concentration of total saturated fatty acids (SFAs) of 31.3% and 16.0%, MUFAs of 17.7% and 57.2%, and PUFAs of 51.1% and 26.8%, respectively, with the best oleic/linoleic ratio among all the analyzed lines (Table 1). Moreover, *CcFAD2.2*KO2 transgenic line showed an oleic/linoleic ratio that was about six times higher than that of the control and *CcSAD*OE6 lines. Nevertheless, regarding the lipid yield, no statistically significant difference was detected among analyzed transgenic lines. All lines, including WT and EV control ones, showed a lipid content ranging from 1.9 to 3.6% (w/w), as reported in Supplementary Table 4.

**TABLE 1:** Fatty acid content was detected by gain control (GC)-FID in untransformed (WT) and transgenic *CcSAD*OE6 and *CcFAD2.2* KO2 *Cynara cardunculus* calli lines.

Fatty acids	WT	*CcSAD*OE6	*CcFAD*2.2KO2
Palmitic Acid	29.7 ± 0.5*^a^*	24.8 ± 2.1*^a^*	12.8 ± 0.5*^b^*
Palmitoleic Acid	0.5 ± 0.3*^a^*	n.d.	n.d.
Cis-10-Heptadecenoic Acid	3.9 ± 0.6*^a^*	0.4 ± 0.1*^b^*	0.4 ± 0.2*^b^*
Stearic Acid	9.2 ± 0.5*^a^*	5.6 ± 0.4*^b^*	2.7 ± 0.2*^c^*
Oleic Acid	17.7 ± 0.3*^b^*	17.3 ± 1.1*^b^*	56.7 ± 0.8*^a^*
Linoleic Acid	30.4 ± 0.7*^b^*	42.5 ± 1.9*^a^*	18.6 ± 0.2*^c^*
Arachidic Acid	*n*.*d*.	0.9 ± 0.1*^a^*	0.5 ± 0.2*^a^*
Linolenic Acid	8.6 ± 0.4*^a^*	8.6 ± 0.7*^a^*	8.1 ± 0.3*^a^*
SFAs	38.8 ± 1.1*^a^*	31.3 ± 1.9*^a^*	16.0 ± 0.9*^b^*
MUFAs	22.2 ± 0.9*^b^*	17.7 ± 1.2*^c^*	57.2 ± 1.0*^a^*
PUFAs	39.0 ± 0.8*^b^*	51.1 ± 2.4*^a^*	26.8 ± 0.5*^c^*
C18:1/C18:2	0.58 ± 0.10*^b^*	0.41 ± 1.8*^b^*	3.04 ± 0.11*^a^*

*Values are expressed as % of the total; SFA, saturated fatty acids; MUFAs, monounsaturated fatty acids; PUFAs, polyunsaturated fatty acids; C18:1/C18:2 oleic/linoleic acid ratio. Each value represents the mean value ± SD (n = 3). Different letters in the same row indicate statistically significant differences through ANOVA. Statistical significance was defined as p < 0.05, using Tukey’s post hoc test for mean separation. n.d., not detected.*

#### Polyphenols Profile Changes in *CcSADOE* and *CcFAD2.2KO* Lines

The qualitative-quantitative profile of polyphenolic compounds makes a significant contribution to the total bioactivity of plant materials and was therefore analyzed at 28 days of growth in WT and *CcSAD*OE6 and *CcFAD2.2*KO2 lines, selected as the best transformants in terms of modified fatty acid profile, using a specific non-target UHPLC-Q-Orbitrap HRMS analysis (Supplementary Table 5). Typical full-scan chromatograms and the specifications of the observed peaks are reported in Supplementary Figure 4. Among the investigated polyphenolic compounds, 5 compounds were unambiguously quantified by comparing with reference standards. For the compounds without available references, the structures were presumed based on a high-accuracy analysis of deprotonated precursors and confirmed by MS^2^ experiments. On average, WT calli had a total polyphenolic content of 3,652 μg/g DW, while transgenic lines are characterized by a significant increase, larger in *CcSAD*OE6 (+ 28%) than *CcFAD2.2*KO2 calli lines (+ 13%). In both transgenic lines, hydroxycinnamic acids, the main phenolic compounds in *C. cardunculus*, account for the largest fraction. Indeed, a higher accumulation was detected for 3,4 diCQA and 1,5 diCQA compared with WT lines. Among the major bioactive compounds, CGA (565.903 μg/g DW in WT) was not significantly changed in *CcSAD*OE6 (572 μg/g DW) and *CcFAD2.2*KO2 (521 μg/g DW) lines, whereas isochlorogenic acid and cynarin showed the highest increase in both transgenic lines. Feruloyl quinic acids (3-FQA and 5-FQA) were also found to be prevalent in transgenic lines compared with the WT calli, considering that the sum of their concentration was 29.63 μg/g DW in the WT, while *CcSAD*OE and *CcFAD2.2*KO2 lines reached values of 109.18 and 119.37 μg/g DW, respectively. A similar trend was observed for flavones, luteolin-rutinoside, and myricetin, significantly more represented in transgenic lines than in the WT. Ferulic acid, luteolin-glucoside, and coumaric acid showed higher levels in *CcSAD*OE lines with concentrations of 8.88, 0.12, and 50.64 μg/g DW, respectively, while no significant differences were found between WT and *CcFAD2.2*KO2 silenced lines. Finally, as regards quercetin glucoside, diosmine, and hydroxybenzoic acid, no substantial differences were observed between the *SAD*-overexpressing, *FAD2.2*-silenced, and WT callus lines (Table 2).

**TABLE 2:** Biochemical characterization of *C. cardunculus* WT and transgenic calli lines, overexpressing a *CcSAD* gene (*CcSADOE6)* or silenced for a *CcFAD2.2* gene (*CcFADKO2*).

Sample	luteolin-rutinoside	3-O-CQA	ferulic acid	3,4-diCQA*	1,5-diCQA	luteolin-glucoside	quercetin-glucoside	coumaric acid	3-FQA	5-FQA**	Diosmin	hydroxy-benzoic acid	myricetin
**ppm (μg g^–1^ DW)**													
Wild type	0.063 ± 0.001a	565.903 ± 25.837a	6.991 ± 0.480a	1455.677 ± 311.999a	443.230 ± 104.583a	0.079 ± 0.007a	0.056 ± 0.002a	36.355 ± 2.471a	18.934 ± 1.692a	10.700 ± 0.342a	0.087 ± 0.004a	177.955 ± 20.040a	0.037 ± 0.003a
*CcSAD*OE6	0.302 ± 0.129b	572.048 ± 125.951b	8.886 ± 0.532b	2544.272 ± 89.776b	1223.165 ± 75.417b	0.119 ± 0.003b	0.030 ± 0.002b	50.636 ± 4.353b	69.280 ± 8.159b	39.906 ± 4.469b	0.077 ± 0.022a	150.490 ± 16.233a	0.288 ± 0.049b
*CcFAD2.*2KO2	0.109 ± 0.004c	521.152 ± 40.464a	5.625 ± 0.429c	2266.755 ± 119.091c	974.566 ± 159.536c	0.073 ± 0.011a	0.083 ± 0.004c	38.622 ± 2.090c	80.758 ± 5.643c	38.608 ± 9.378c	0.028 ± 0.002b	203.912 ± 9.912b	0.109 ± 0.008c

Polyphenols content detected by HRMS-Orbitrap: values are expressed in ppm = μg/g dry weight, DW. 1,5-DiCQA, 1,5-dicaffeoylquinic acid (cynarin); 3,4-DiCQA, 3,4-dicaffeoyl quinic acid; 5-FQA, 5-feruloyl quinic acid; 3-FQA,3-feruloyl quinic acid; 3-CQA,3-caffeoyl quinic acid (chlorogenic acid, CGA). The data are presented as mean ± SD (n = 3). Different letters indicate statistically significant differences (p < 0.05). *Concentration for this compound was calculated using the calibration curve of available standard 1,5-diCQA. **Concentration for this compound was calculated using the calibration curve of available standard 3-FQA.

#### Can Metabolic Changes Modify Calli Growth?

**FIGURE 5:**
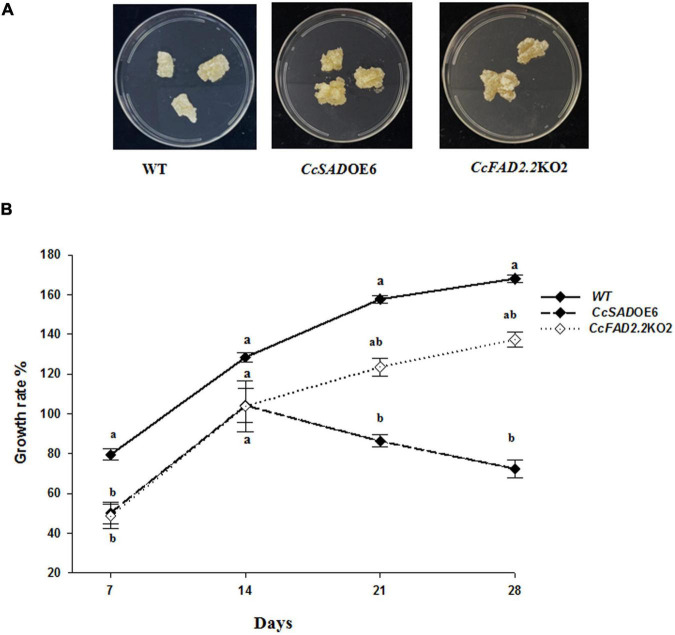
Phenotype and growth of cardoon lines. **(A)** Phenotype of *C. cardunculus* wild type (WT), SAD6 overexpressing line (*CcSADO*E6), and FAD2 silenced line (*CcFAD2.2*KO2). **(B)** Growth rate measurements of WT, *Cc*SADOE6, and *Cc*FAD2.2KO2 transformed cardoon lines at 7-, 14-, 21-, and 28-day culture on fresh media. The data are presented as mean ± SD/10 (*n* = 16). Different letters indicate statistical differences (*p* < 0.05).

To verify whether the observed metabolic changes in fatty acid and phenylpropanoids metabolic flux might influence the growth of transgenic lines, the dynamics of biomass accumulation in transgenic cell lines were measured. To this end, changes in the fresh weight of WT, EV controls, *CcSAD*OE, and *CcFAD2.2*KO transgenic lines were analyzed at 7, 14, 21, and 28 days of cultivation on a GB5 growth medium (Supplementary Figures 5A,B). Over the cultivation period, the five independent *CcSAD*OE transformant lines showed either a growth similar to untransformed calli or a moderate growth reduction at later times (21 and 28 days) of observation. In particular, *CcSAD*OE5 and *CcSAD*OE14 showed a growth comparable to controls at all time points, whereas *CcSAD*OE6 and *CcSAD*OE7 did not increase their biomass at 21 and 28 days and *CcSAD*OE9 even showed growth contraction at 28 days (Supplementary Figure 5A). Instead for *CcFAD2.2*KO calli, all the five independent transgenic lines showed a uniform growth behavior, with a growth lag during the first 7–21 days and fresh biomass similar or slightly increased at 28 days, as is the case of the *CcFAD2.2*KO3 and *CcFAD2.2*KO13 lines, as compared with WT and EV control lines (Supplementary Figure 5B). Regarding control lines, growth confirmed a lack of significant differences between WT and EV calli (Supplementary Figures 5A,B), similar to what was found for molecular and biochemical data. By visual observation, WT and transgenic callus types did not highlight morphological differences, by way of example besides a similar yellowish callus color for WT and *CcSAD*OE6 calli compared with a pale grayish yellow appearance for Cc*FAD2.2KO2* calli (Figure 5A). To better characterize *CcSAD*OE6 and *CcFADKO2* lines, showing the best modified fatty acid profile and improved polyphenol content, a growth rate analysis was performed over 28 days (Figure 5B). In the first week, cells of both transgenic lines were dividing rather slower than WT cells. During the period of 7/14 days, untransformed WT calli increased their weight by about 80%, whereas *CcSAD*OE6 and *CcFAD2.2*KO2 calli lines grew 30% less but recovered their growth by doubling their initial biomass. Main changes in growth showed up from 14 to 28 days, when the *CcFAD2.2*KO2 line maintained a growth increase of about 20% from days 14 to 21 and 10% from days 21 to 28, not statistically different from the WT, whereas *CcSAD*OE6 calli showed a −20% and −10% growth reduction at each time point, although not statistically different from *CcFAD2.2*KO2. Overall, upon 28 days of growth, *CcSAD*OE6 calli showed significantly reduced growth compared with WT, whereas the *CcFAD2.2*KO2 line behaved more similarly to the untransformed control.

## Discussion

Increasing attention of the industry to the use of oilseed crops-derived raw materials has prompted genetic engineering approaches for modifying their fatty acid composition to produce higher oil content or better oil characteristics (Rahman and de Jiménez, 2016; Villanueva-Mejia and Alvarez, 2017). However, restrictions to the cultivation of GM plants in several countries and recalcitrance of numerous relevant oleaginous crops, including cardoon, to regeneration limit this approach. In this context, bioengineered cell factories of oilseed species to produce high oleic acid along with other valuable molecules can be an attractive, renewable, and sustainable manner alternative platform to plant cultivation (Bhati et al., 2021). GM approaches could also be directed to improve the efficiency of WT callus cultures as a potential source for plant oils (Chandran et al., 2020), since it was observed, for example, in *Jatropha curcas*, that they have reduced the percentage of stearic and linoleic acids (C18) in calli than plant seed oil (Costa et al., 2015).

In our approach, transgenic calli with increased oleic acid content were obtained through metabolic engineering of fatty acid biosynthesis. To select key fatty acid biosynthetic genes as targets, we investigated the abundance and evolutionary pattern of *CcSAD* and *CcFAD2* (coding for committed enzymes in oleic and linoleic acid biosynthesis genes) in the *C. cardunculus* genome. Four and sixteen isoforms for *SAD* and *FAD2* genes were found, respectively, none of which were previously characterized in cardoon. The four SAD isoforms are highly homologous, also compared with other species, and possess the same and unique fatty acid desaturase 2 domain (pfam03405) as well as the same motif distribution (Figure 2A). Nevertheless, lack of the SAD motif-6 in the multicellular chlorophyte alga *Volvox carteri* supports the possibility that these proteins arose by independent gene duplication events in green algae and higher plants (Chi et al., 2008). These features suggest that cardoon SAD proteins can all catalyze the desaturation of C18:0-ACP to C18:1-ACP and are probably functionally indistinguishable, as observed in other species (Kachroo et al., 2006; Li et al., 2015; Zhang et al., 2015). Therefore, we selected the *SAD* isoform V2_02g007570.1.01 as the target for overexpression, based on its high sequence homology to other functionally characterized *SAD*s (Li et al., 2014; Dmitriev et al., 2020).

Regarding the *FAD* genes, phylogenetic analysis revealed the occurrence of three main groups, corresponding to the three distinct tissue-specific types (Figure 2B) also found in other species (Dar et al., 2017). Our analysis corroborates the reported huge separation between branches of *FAD2* (including the house-keeping and seed-type FAD2) and the plastidial *FAD6* suggesting that they diverged during early gene evolution (Dar et al., 2017). Structural analysis also confirms the conserved evolution of the house-keeping and seed-type FAD2, supporting the group classification, although the motif’s positional distribution among the sequences is not always conserved in members of the *CcFAD2* seed-type subfamily. We found 14 isoforms of the seed-type *FAD2.1*, whose chromosomal location suggests they might have evolved from gene family expansion, since at least four pairs clustered together on chromosomes 2, 13, and 15 (Figure 2B and Supplementary Figure 1), which can be interpreted as evidence of gene duplication. On the contrary, *FAD6* and *FAD2.2* were found in only one copy in the cardoon transcriptome, and the strong conservation of their motifs in most analyzed species suggests their crucial involvement in protein and enzyme functions. As shown in the phylogenetic tree (Figure 2B), those FAD desaturases grouped with algae desaturases also fell into the two distinct microsomal (FAD 2.2) and chloroplastic (FAD6) subfamilies, thus indicating a common evolution for plant and eukaryotic algae (Chi et al., 2008). So far in cardoon varieties, among which “Spagnolo” used in this study to generate callus, the expression profile of *FAD2.2* could not allow identifying this gene as solely associated with linoleic acid content (Graziani et al., 2020) although Hernández and colleagues (Hernández et al., 2009) identified the *FAD2.2* gene as the major gene responsible for the synthesis of linoleic acid in olive. Nevertheless, as in other oleaginous plants, its expression is in line with constitutive gene functionality, and therefore, *CcFAD2.2* (V2_05g005480.1.01) was selected as a target for RNAi.

Analysis of fatty acid content and transcriptional activities of *CcSAD* and *CcFAD2.2* genes confirmed that fatty acid biosynthesis is properly working in cardoon calli, which could therefore be used for biotechnological manipulation and development of an improved platform for the production of oleic acid and other valuable molecules. Calli revealed a similar profile and ratio of fatty acids of the leaf tissue (Graziani et al., 2020; Figure 3A), although with reduced accumulation of oleic and linoleic acids, mirrored by a ten-fold reduction of transcriptional activity of *SAD* and *FAD2.2*-coding genes (Figure 3B). These results were expected since in plants desaturation of fatty acids is influenced by light (Murphy and Stumpf, 1979), whereas most cell cultures including ours grow in the dark. In dark growing soybean cells, the lower level of fatty acids desaturation was associated with light-dependent transcriptional regulation of *FAD* genes (Collados et al., 2006). Based on molecular and metabolic characteristics detected in cardoon calli, an *Agrobacterium*-mediated metabolic engineering strategy seemed useful to improve oleic acid content in cardoon. The obtained transformed calli populations, *CcSAD* overexpressing, and *CcFAD2.2* knocked out lines, based on their respective transgene transcript abundance, showed various degrees of expression compared with WT and empty vector controls (Figures 4A,B). Since the constitutive introduction of foreign DNA may cause silencing side effects due to excessive transcription of the engineered gene (Gazzani et al., 2004), and effective silencing is not strictly linked to the lowest transcript levels (El-Sappah et al., 2021), the selection of the most efficient independent transformants was made based on fatty acid content. In both gene manipulation strategies, a modification of the fatty acids flux in favor of oleic acid production was positively reached, and this was more evident for FAD silenced lines (Supplementary Figures 3A,B). *CcSAD*OE6 and *CcFAD2.2*KO2 lines showed the most notable effects on oleic acid synthesis, accompanied in both cases by a significant reduction in stearic acid, indicating FA as the main precursor for the increased MUFAs and PUFAs flux. In detail, *CcSAD*OE6 line seemed to address the metabolic flux toward an increase of linoleic acid (42.5%) without impact on the oleic acid level that resulted comparable with WT (18%). Instead, for the *CcFAD2.2*KO2 line, oleic acid raised its content to almost 60% at the expense of linoleic acid accumulation (Table 1). A wide literature confirmed the key role of *SAD* or *FAD2* genes’ manipulation to modify the MUFA/PUFAs ratio in several oilseed crops. Since SAD catalyzes the desaturation of 18: 0 to 18: 1Δ9, it determines the ratio of saturated to unsaturated fatty acids (Smith et al., 2013). Our results confirmed the trend reported in both maize and *Arabidopsis* that showed reduced stearic acid content as the main effect of SAD overexpression, while the oleic acid level was comparable with the control (Du et al., 2016). However, the metabolic flux was not directed toward a significant increase of linoleic acid, as occurred in the *Cc*SADOE6 line. Similarly, higher linoleic and oleic acid content detected in *Chlamydomonas reinhardtii* transgenic lines overexpressing the *SAD* gene *Cr*FAB2 partially support our data (Hwangbo et al., 2014). The ability of *CcSAD*OE6 line to accumulate linoleic acid is of great interest because of several applications of this fatty acid for food and non-food uses (Abdullah et al., 2016; Shinn et al., 2017).

RNAi-mediated silencing of *FAD2* genes was successfully used to increase oleic acid yield in flax and safflower plants (Chen et al., 2015; Wood et al., 2018). Similarly, *CcFAD2.2* gene silencing of *C. cardunculus* calli determined higher oleic content at the expense of linoleic acid (Table 1 and Supplementary Figure 3B), supporting the reported role of the encoded enzyme in modifying the oleic/linoleic acid ratio (Hernández et al., 2021). Our results demonstrate that cardoon callus cultures could be a promising alternative, extendable to other valuable Asteraceae species, to plant-based high oleic platforms, which bypasses the drawbacks of limitations to GM plant cultivation and the concerns about diverting land use from food and feed production, thus contributing to meet the exponentially growing demand of oils from renewable sources as chemical raw materials and biofuels. Our silencing approach also meets the needs of the agro-industrial market, since high oleic oils with reduced PUFAs have improved oxidative stability, being less susceptible to autoxidation and polymerization reactions (Mancini et al., 2019), which is important not only for food use but also for industrial applications. Therefore, the composition of the oil from the engineered *C. cardunculus* calli makes it promising as a raw material for producing cosmetics, biodiesel, lubricants, bioplastics, and other valuable materials (Barracosa et al., 2019; Turco et al., 2019).

Besides oil, cardoon tissues and organs, in particular, leaves, are also rich in bioactive compounds that can find many applications in the pharmaceutical and nutraceutical industries (Petropoulos et al., 2019), such as flavonoids (i.e., luteolin and apigenin), and chlorogenic and caffeoylquinic acids (Fratianni et al., 2007; Graziani et al., 2020), with important antioxidant functions. Wild-type cardoon calli also reflected this metabolic scenario. Our metabolic engineering strategy exerted a positive effect also on the polyphenolic profiles of *CcSAD*OE6 and *CcFAD*2.2KO2 transformed lines, which showed a statistically significant accumulation of isochlorogenic acid and cynarin, as well as feruloyl quinic acids (3-FQA and 5-FQA) and flavones, compared with untransformed calli. In particular, *CcSAD*OE6 showed the highest accumulation of polyphenols. The improved phytochemical composition of the transgenic lines, both in terms of fatty acids and polyphenol species, further sustains a successful use of *C. cardunculus* calli for the development of innovative bioproducts from primary and secondary metabolism, in a true biorefinery perspective.

Since an important requisite for efficient biomolecules production is the maintenance of constant growth and high biomass yield, we analyzed the effects of genetic transformation on calli growth of *CcSAD* overexpressing and *CcFAD*2.2 silenced lines. Overall, all the analyzed transformed lines did not suffer from negative perturbation of growth and biomass as indicated by the visual appearance of transformed calli and by the absence of relevant changes in fresh weight of five independent transgenic lines for each construct (Supplementary Figures 5A,B). The growth rate analysis of *CcSAD*OE6 and *CcFAD*2.2KO2 lines over 28 days (Figure 5B) indicated a slight growth rate reduction of the SAD overexpressing line at later stages. In *Arabidopsis*, a negative correlation between the growth rate of cell cultures and the level of PUFA (18:2 + 18:3) was reported (Meï et al., 2015). Accordingly, we could speculate that the negative effect on cell growth in the *CcSAD*OE6 line may be ascribed to its higher linoleic acid content. Our results are also supported by data of Paolo et al. (2021), indicating a positive correlation between faster growth rate and higher levels of oleic acid (C18:1) at the expense of PUFA levels in AtMYB4oe cardoon calli lines. Nevertheless, we cannot rule out that also the perturbation of the phenylpropanoids metabolism revealed in *CcSAD*OE6 overexpressing and *CcFAD*2.2KO2 silenced lines might influence the dynamics of biomass accumulation in the transformants. In the *CcSAD*OE6 line, the highest accumulation of polyphenols (Table 2) could contribute to the observed growth reduction, since an increase in specialized metabolites accumulation in plants can be connected to pleiotropic effects on growth (Sestari and Campos, 2021). In the latter case, a trade-off mechanism could be established between primary and hence biomass production and secondary metabolism (i.e., synthesis of defense compounds) (Lattanzio et al., 2018). In a biorefinery perspective, higher production of valuable specialized metabolites could compensate for a moderate growth reduction of the cultures. However, preliminary results in pilot bioreactors show higher biomass productivity in liquid than in solid medium for *CcSAD*6OE, while confirming the growth trend for *CcFAD2.2*KO2 (L. Langellotti—University of Naples Federico II, personal communication, March 2022). In light of these findings, we believe that our transgenic cardoon lines have an attractive potential as innovative biorefinery routes for the portfolio of products that can be obtained.

## Conclusion

In this study, we identified *SAD* and *FAD* genes in the cardoon genome and carried out phylogenetic analysis to determine the relationships among them. To the best of our knowledge, this is the first study that has identified and characterized two putative genes (*CcSAD1* and *CcFAD2.2*) involved in oleic acid and linoleic acid formation in *C. cardunculus*. Moreover, our biotechnological approach allowed the production and selection of the two lines *CcSAD*OE6 and *CcFAD2.2*KO2 for their ability to direct the flux of fatty acid biosynthesis toward higher linolenic or oleic content, thus allowing the diversification of oil profiles in calli lines to expand market opportunities for cardoon products. The above transgenic lines also showed improved phytochemical composition, meeting the strong demand from the industry for phenolic acids as valuable precursors of other significant bioactive molecules, which are needed on a regular basis for the therapeutic, cosmetics, food, and green chemistry sectors. Therefore, the outcome of our investigations poses the basis for the development of an engineered cell biofactory ensuring good biomass production and accumulation of MUFAs and PUFAs along with other valuable molecules, as an attractive alternative platform to plant cultivation for the renewable and sustainable production of valuable bioproducts.

## Data Availability Statement

The original contributions presented in the study are included in the article/Supplementary Material. Further inquiries can be directed to the corresponding author.

## Author Contributions

EC, MDP, TD, and MT did the conceptualization. EC, RD’A, TD, GG, and AA performed the methodology. EC, MDP, TD, AR, GG, and AA investigated the data and carried out the formal analysis. EC, MDP, TD, GG, and AA wrote the original draft preparation. EC, MDP, TD, FS, DP, FL, MDP, AA, and MT wrote, reviewed, and edited the manuscript. TD, MT, AR, and RR supervised the data. MT and FS carried out the project administration. AR, MT, and FS carried out the funding acquisition. All authors have read and agreed to the published version of the manuscript.

## Conflict of Interest

The authors declare that the research was conducted in the absence of any commercial or financial relationships that could be construed as a potential conflict of interest.

## Publisher’s Note

All claims expressed in this article are solely those of the authors and do not necessarily represent those of their affiliated organizations, or those of the publisher, the editors and the reviewers. Any product that may be evaluated in this article, or claim that may be made by its manufacturer, is not guaranteed or endorsed by the publisher.

## Footnotes

1
http://www.artichokegenome.unito.it/

2
http://visualization.ritchielab.psu.edu/phenograms/plot

3
https://www.genome.jp/tools-bin/clustalw

4
http://meme-suite.org/tools/meme
