# Supplementary material for: Development of a High Oleic Cardoon Cell Culture Platform by SAD Overexpression and RNAi-Mediated FAD2.2 Silencing
Source: Front Plant Sci. 2022 Jun 20;13:913374. doi: 10.3389/fpls.2022.913374 (PMC9285897; doi:10.3389/fpls.2022.913374)
Supplement: Supplementary file 1 [file Data_Sheet_1.docx]

Supplementary Material


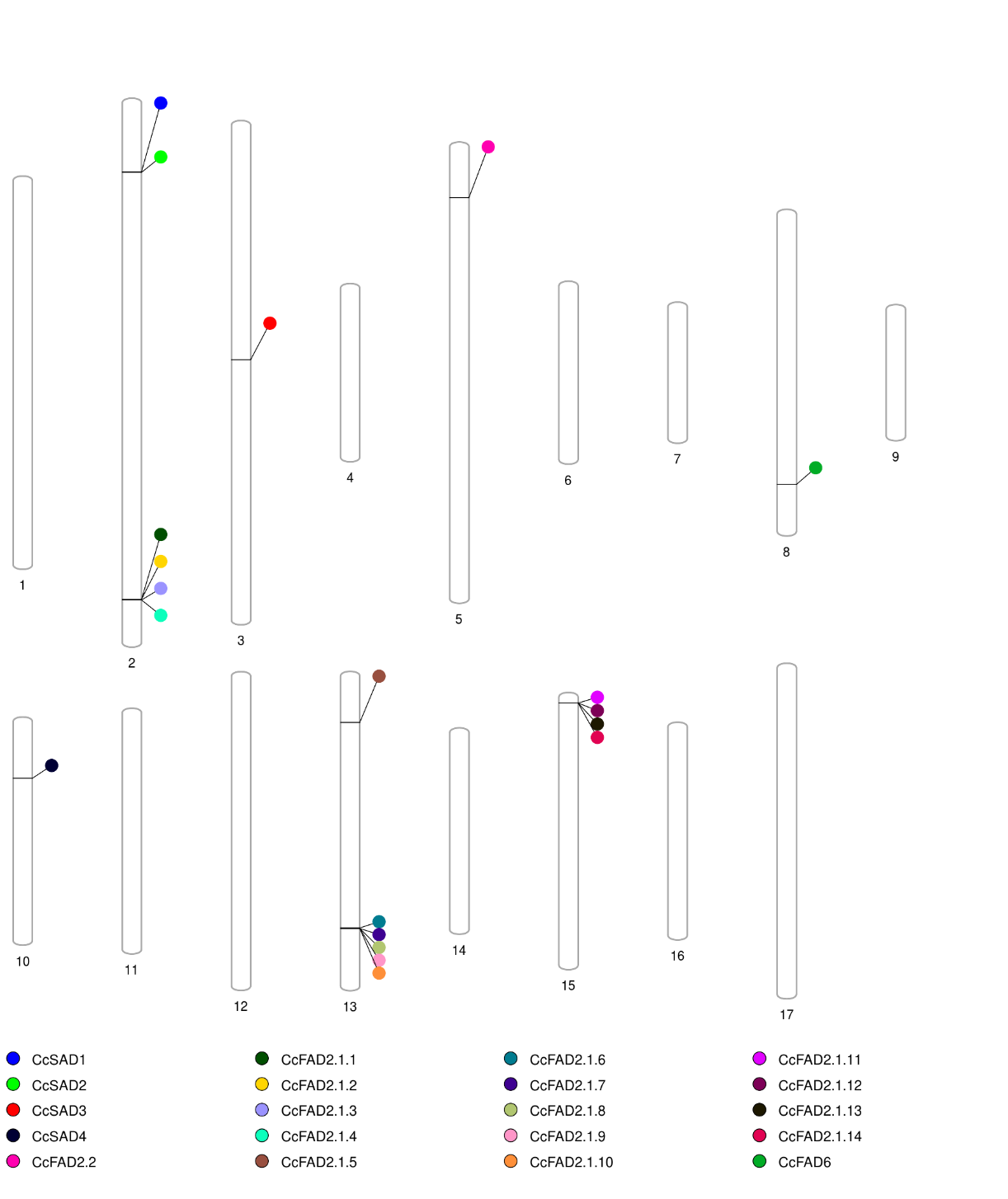


**Supplementary Figure 1** Phenogram represents the chromosomal location of *CcSAD* and *CcFAD2* genes on each chromosome of *Cynara cardunculus.*

**Supplementary Table S1** List of the identified *CcSAD* and *Cc*F*AD2* genes and their characteristics in the *Cynara cardunculus* genome.

|  | | | | | | | | |
| --- | --- | --- | --- | --- | --- | --- | --- | --- |
| **Gene ID** | **Gene name** | **Chromosome** | **Amino acid residues** | **MW (Da)** | **pI** | **II** | **AI** | **GRAVY** |
| V2_02g007570.1.01 | *CcSAD1* | chr02 | 382 | 43400.39 | 6.71 | 38.22 | 81.47 | -0.297 |
| V2_02g007620.1.01 | *CcSAD2* | chr02 | 382 | 43596.92 | 6.44 | 40.27 | 86.60 | -0.310 |
| V2_03g010860.1.01 | *CcSAD3* | chr03 | 396 | 44724.35 | 5.81 | 40.28 | 82.02 | -0.281 |
| V2_10g007430.1.01 | *CcSAD4* | chr10 | 396 | 44998.31 | 6.29 | 37.03 | 79.07 | -0.425 |
| V2_02g021840.1.01 | *CcFAD2.1.1* | chr02 | 378 | 43676.50 | 8.70 | 36.24 | 95.19 | -0.011 |
| V2_02g021850.1.01 | *CcFAD2.1.2* | chr02 | 499 | 57439.61 | 8.08 | 36.57 | 101.58 | 0.110 |
| V2_02g021860.1.01 | *CcFAD2.1.3* | chr02 | 378 | 43758.52 | 8.69 | 36.07 | 93.86 | -0.034 |
| V2_02g021870.1.01 | *CcFAD2.1.4* | chr02 | 1036 | 119703.45 | 8.91 | 32.06 | 95.88 | 0.014 |
| V2_05g005480.1.01 | *CcFAD2.2* | chr05 | 774 | 87495.49 | 6.26 | 40.63 | 93.17 | -0.095 |
| V2_08g011560.1.01 | *CcFAD6* | chr08 | 433 | 49979.26 | 9.41 | 47.65 | 89.24 | -0.093 |
| V2_13g003900.1.01 | *CcFAD2.1.5* | chr13 | 376 | 43837.29 | 6.73 | 37.40 | 85.29 | -0.112 |
| V2_13g014320.1.01 | *CcFAD2.1.6* | chr13 | 478 | 55331.62 | 7.14 | 35.82 | 89.77 | -0.071 |
| V2_13g014330.1.01 | *CcFAD2.1.7* | chr13 | 374 | 44003.51 | 7.08 | 33.44 | 84.47 | -0.104 |
| V2_13g014340.1.01 | *CcFAD2.1.8* | chr13 | 1611 | 187397.63 | 7.21 | 32.98 | 80.81 | -0.105 |
| V2_13g014350.1.01 | *CcFAD2.1.9* | chr13 | 376 | 43799.15 | 6.90 | 33.49 | 80.16 | -0.111 |
| V2_13g014360.1.01 | *CcFAD2.1.10* | chr13 | 764 | 88739.11 | 7.89 | 37.53 | 83.99 | 0.004 |
| V2_15g000720.1.01 | *CcFAD2.1.11* | chr15 | 702 | 81498.34 | 6.74 | 37.78 | 84.33 | -0.098 |
| V2_15g000730.1.01 | *CcFAD2.1.12* | chr15 | 435 | 50098.77 | 6.49 | 35.70 | 85.77 | -0.027 |
| V2_15g000740.1.01 | *CcFAD2.1.13* | chr15 | 380 | 44232.54 | 7.80 | 32.75 | 88.74 | 0.034 |
| V2_15g000750.1.01 | *CcFAD2.1.14* | chr15 | 475 | 54006.84 | 9.43 | 36.18 | 80.65 | -0.137 |

Molecular weight (MW), Isoelectric point (pI), Instability index (II), Aliphatic index (AI), Grand average of hydropathicity (GRAVY)

| **Organism name** | **Protein ID** | **Protein name** |
| --- | --- | --- |
| *Cynara cardunculus* | V2_02g007570.1.01 | CcSAD1 |
| *Cynara cardunculus* | V2_02g007620.1.01 | CcSAD2 |
| *Cynara cardunculus* | V2_03g010860.1.01 | CcSAD3 |
| *Cynara cardunculus* | V2_10g007430.1.01 | CcSAD4 |
| *Lupinus luteus* | AAD28287 | LlSAD |
| *Medicago truncatula* | XP_013466413 | MtSAD |
| *Cicer arietinum* | XP_004498381 | CaSAD |
| *Lactuca sativa* | XP_023729196 | LsSAD |
| *Olea europaea* | XP_022879085 | OeSAD |
| *Pistacia vera* | XP_031259402 | PvSAD |
| *Theobroma cacao* | AKF42363 | TcSAD |
| *Ricinus communis* | NP_001310674 | RcSAD |
| *Jatropha curcas* | XP_012066083 | JcSAD |
| *Manihot esculenta* | XP_021603146 | MeSAD |
| *Volvox carteri* | XM_002949594.1 | VcSAD |
| *Volvox carteri* | XP_002955859.1 | VcFAD2 |
| *Brassica napus* | CAA62578.1 | BnFAD2.2 |
| *Brassica carinata* | AAD19742.1 | BcFAD2.2 |
| *Brassica juncea* | ABR27357.1 | BjFAD2.2 |
| *Brassica rapa* | CAD30827.1 | BrFAD2.2 |
| *Brassica napus* | AAF78778.1 | BnFAD2.2 |
| *Arabidopsis thaliana* | AAA32782.1 | AtFAD2.2 |
| *Glycine max* | CAA71199.1 | GmFAD2.2 |
| *Borago officinalis* | AAC31698.1 | BoFAD2.2 |
| *Calendula officinalis* | AAK26633.1 | CoFAD2.2 |
| *Helianthus annuus* | AAL68982.1 | HaFAD2.2 |
| *Helianthus annuus* | AAL68983.1 | HaFAD2.2 |
| *Vernonia galamensis* | AAF04093.1 | VgFAD2.2 |
| *Vernonia galamensis* | AAF04094.1 | VgFAD2.2 |
| *Cynara cardunculus* | V2_05g005480.1.01 | CcFAD2.2 |
| *Crepis palestina* | CAA76157.1 | CpFAD2.2 |
| *Petroselinum crispum* | AAB80696.1 | PcFAD2.2 |
| *Spinacia oleracea* | BAC22091.1 | SoFAD2.2 |
| *Persea americana* | AAL23676.1 | PaFAD2.2 |
| *Arachis duranensis* | AAF82294.1 | AdFAD2.1 |
| *Glycine max* | AAB00859.1 | GmFAD2.1 |
| *Euphorbia lagascae* | AAS57577.1 | ElFAD2.1 |
| *Vernicia fordii* | AAN87574.1 | VfFAD2.1 |
| *Gossypium hirsutum* | CAA65744.1 | GhFAD2.1 |
| *Punica granatum* | CAD24671.1 | PgFAD2.1 |
| *Sesamum indicum* | AAF80560.1 | SiFAD2.1 |
| *Solanum commersonii* | CAA63432.1 | ScFAD2.1 |
| *Helianthus annuus* | AAL68981.1 | HaFAD2.1 |
| *Cynara cardunculus* | V2_02g021840.1.01 | CcFAD2.1.1 |
| *Cynara cardunculus* | V2_02g021850.1.01 | CcFAD2.1.2 |
| *Cynara cardunculus* | V2_02g021860.1.01 | CcFAD2.1.3 |
| *Cynara cardunculus* | V2_02g021870.1.01 | CcFAD2.1.4 |
| *Cynara cardunculus* | V2_13g003900.1.01 | CcFAD2.1.5 |
| *Cynara cardunculus* | V2_13g014320.1.01 | CcFAD2.1.6 |
| *Cynara cardunculus* | V2_13g014330.1.01 | CcFAD2.1.7 |
| *Cynara cardunculus* | V2_13g014340.1.01 | CcFAD2.1.8 |
| *Cynara cardunculus* | V2_13g014350.1.01 | CcFAD2.1.9 |
| *Cynara cardunculus* | V2_13g014360.1.01 | CcFAD2.1.10 |
| *Cynara cardunculus* | V2_15g000720.1.01 | CcFAD2.1.11 |
| *Cynara cardunculus* | V2_15g000730.1.01 | CcFAD2.1.12 |
| *Cynara cardunculus* | V2_15g000740.1.01 | CcFAD2.1.13 |
| *Cynara cardunculus* | V2_15g000750.1.01 | CcFAD2.1.14 |
| *Volvox carteri* | XP_002949932.1 | VcFAD6 |
| *Arabidopsis thaliana* | AAA92800.1 | AtFAD6 |
| *Brassica napus* | AAA50157.1 | BnFAD6 |
| *Glycine max* | AAA50158.1 | GmFAD6 |
| *Cynara cardunculus* | V2_08g011560.1.01 | CcFAD6 |
| *Spinacia oleracea* | CAA55121.1 | SoFAD2 |

**Supplementary Table S2** List of the species used to construct the phylogenetic trees.


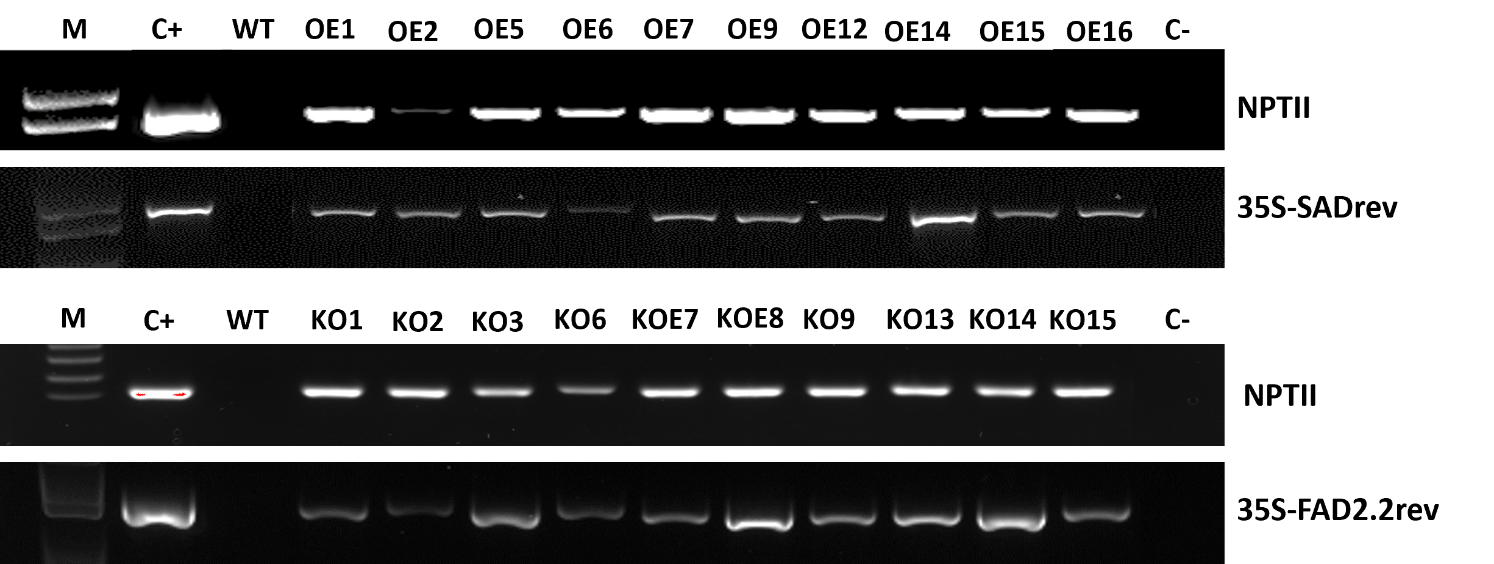


**(B)))**

**(A)**

**Supplementary Figure 2 A-B**. Molecular characterization of ten independent *Cc*SAD overexpressing lines and ten independent *Cc*FAD2.2 silenced lines. (A) PCR amplification of genomic DNA using specific primers for NPTII, and a combination of vector-specific and gene-specific primers (35S and SADrev). M, marker 1Kb DNA ladder; C+, plasmid carrying the exogenous gene; WT, wild type callus; OE, overexpressed transgenic calli; C-, negative control without DNA sample. (B) PCR amplification of genomic DNA using specific primers for NPTII, and a combination of vector-specific and gene-specific primers (35S and FAD2.2rev). M, 1 Kb marker; C+, plasmid carrying the exogenous gene; WT, wild type callus; KO, silenced transgenic calli; C-, negative control without DNA sample.


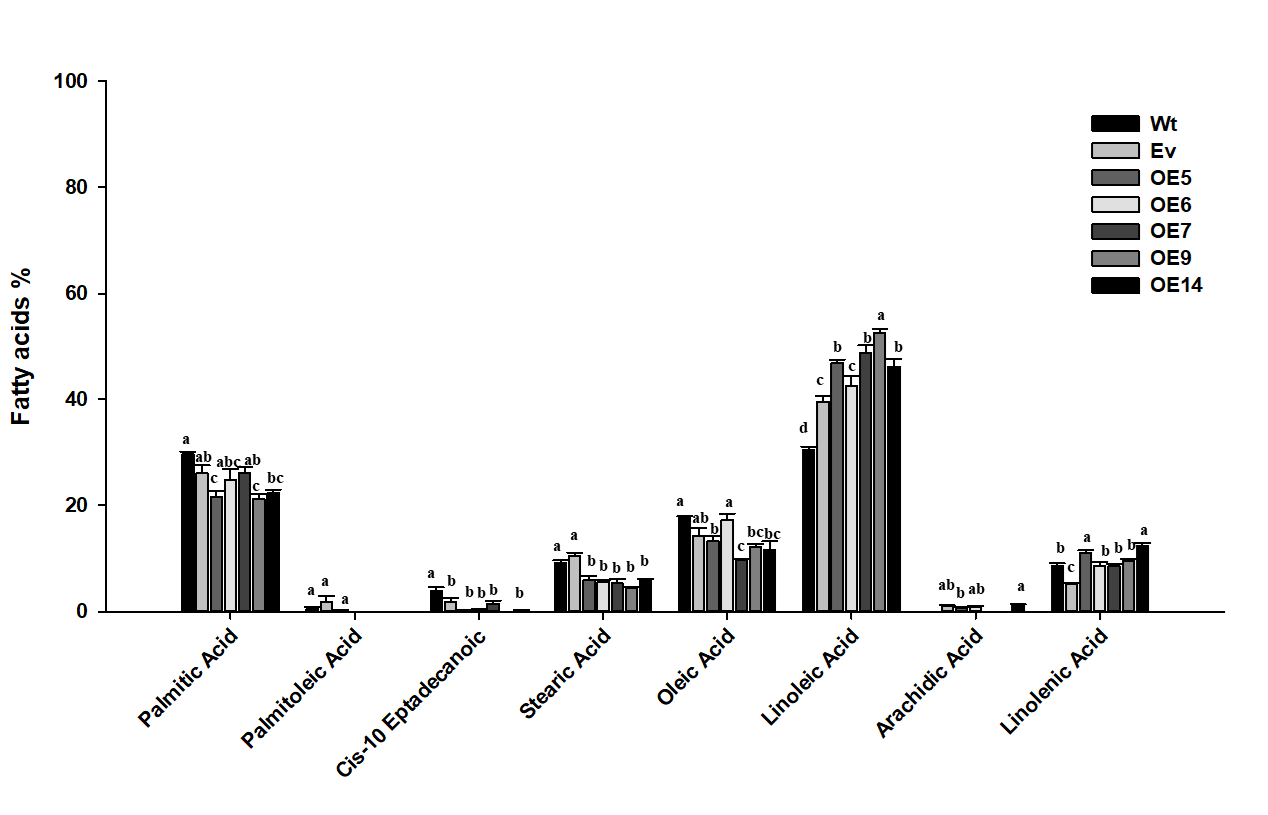


**(A)**


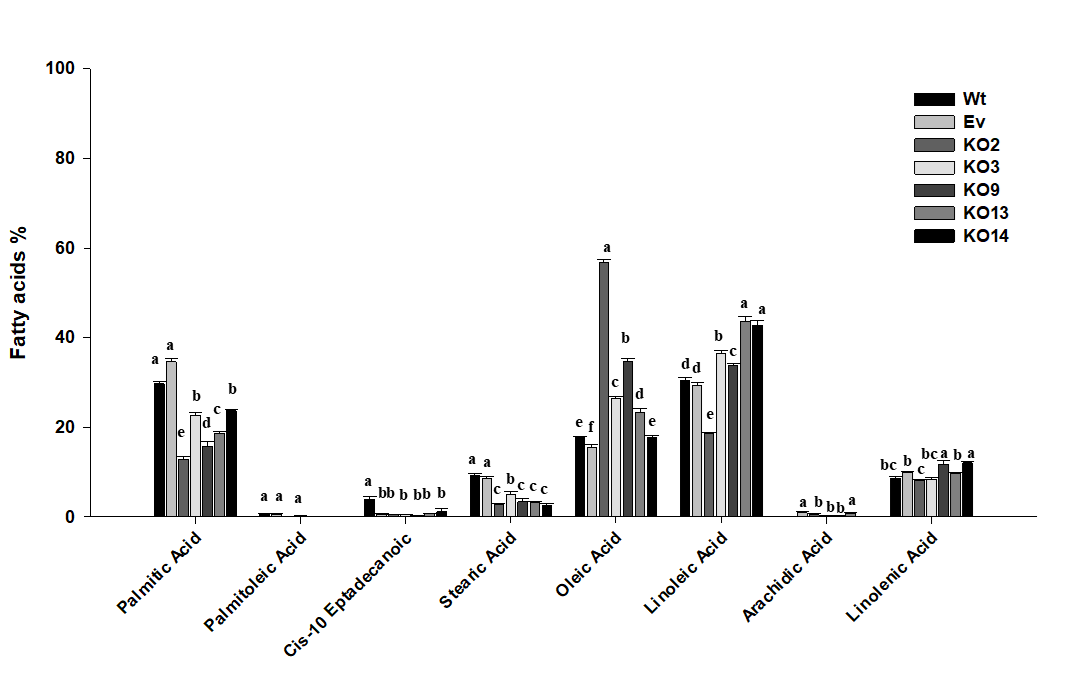


**(B)))**

**Supplementary Figure 3 A-B**. Fatty acid profile of WT, empty vector-transformed, and of five independent transformant lines for *Cc*SAD overexpression and *Cc*FAD2.2 silencing. (A). Fatty acid profile (%) in non agro-inoculated cardoon calli (WT), in empty vector (EV)_transformed and in *Cc*SAD independent overexpressing lines (OE5, OE6, OE7, OE9, OE14). (B) Fatty acid profile (%) in WT cardoon calli, EV-transformed and in *CcFAD2.2*-KO independent silenced lines (KO2, KO3, KO9, KO13, KO14). Data are presented as mean ± SD (*n*=3). Different letters indicate statistically significant differences (*p* < 0.05)

**Supplementary Table S3** List of primers used for analysis.

| **Primer** | **Sequence** |
| --- | --- |
| 35S | CTATCCTTCGCAAGACCCTTC |
| SADseqFor | CACCATGCATTGCATCTCCTCTC |
| SADrev | CTACAGCATGACCTTCTTGTTGAAA |
| FAD2.2seqFor | CACCATGGGTGCAGGTGGACG |
| FAD2.2rev | GTACATGCAAAGGACCCAGGTGAG |
| SADqFor | GATGACCACCACTCACCACC |
| SADqRev | AGGAAAGGGAGGATGGATTG |
| FADqFor | AATCTTCATCTCCGACGCCG |
| FADqRev | GTTCACCACAAGCAACGGTC |
| NPTIIFor | TCAGCCCATTCGCCGCCA |
| NPTIIRev | CTTGCTTGCCGAATATCATGGTGG |
| ActFor | TACTTTCTACAACGAGCTTC |
| ActRev | ACATGATTTGAGTCATCTTC |
| EF For | TGACCCCAGTTTCAACACGG |
| EF Rev | AAGAGGCCATCAGACAAGCC |
| 18SFor | ATGATAACTCGACGGATCGC |
| 18SRev | CTTGGATGTGGTAGCCGTTT |

**Supplementary Table S4**. Fat content analyzed in WT, empty vector-transformed, and of five independent transformant lines for *CcSAD* overexpression and *CcFAD2.2* silencing.

| **Calli lines** | **Fat content (% w/w)** |
| --- | --- |
| WT | 2.3±0.1 ^a^ |
| EV | 2.4±0.3 ^a^ |
| OE5 | 2.1±0.2 ^a^ |
| OE6 | 3.2±0.9 ^a^ |
| OE7 | 2.6±0.4 ^a^ |
| OE9 | 2.7±0.8 ^a^ |
| OE14 | 2.7±0.5 ^a^ |
| KO2 | 3.1±0.2 ^a^ |
| KO3 | 3.6±0.7 ^a^ |
| KO9 | 1.9±0.3 ^a^ |
| KO13 | 2.5±0.6 ^a^ |
| KO14 | 2.7±0.3 ^a^ |
|  |  |


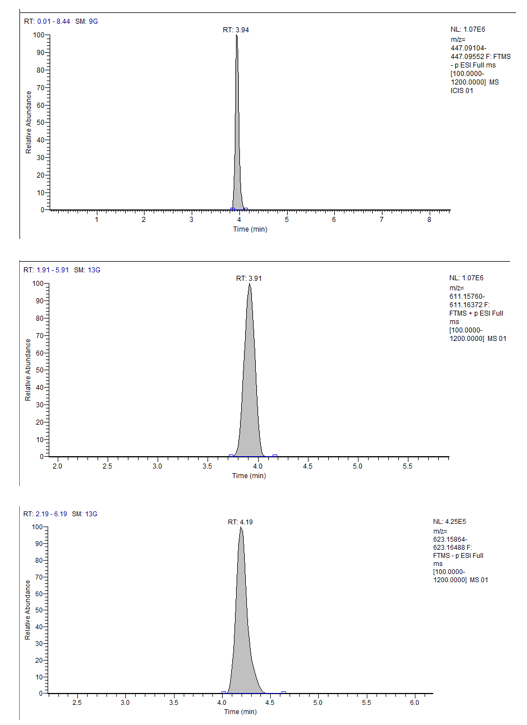


Luteolin-rutinoside

3-O-CQA

Ferulic acid


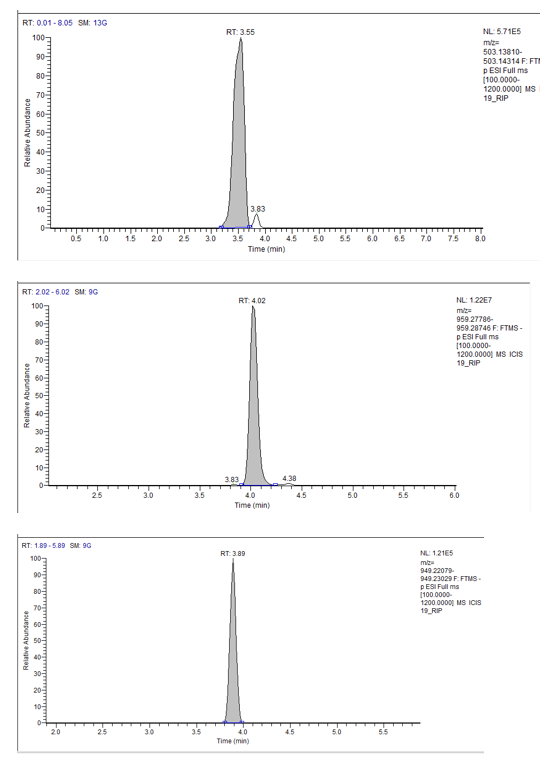


Luteolin-glucoside

1,5-diCQ

3,4-diCQA


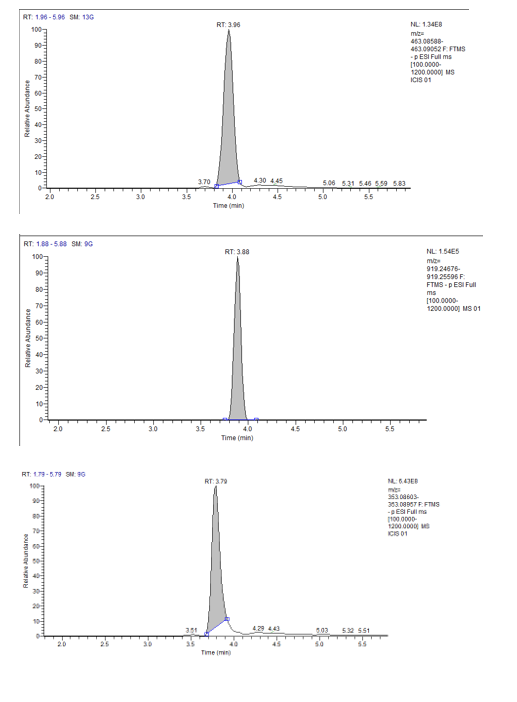


Quercetin-glucoside

coumaric acid

3-FQA


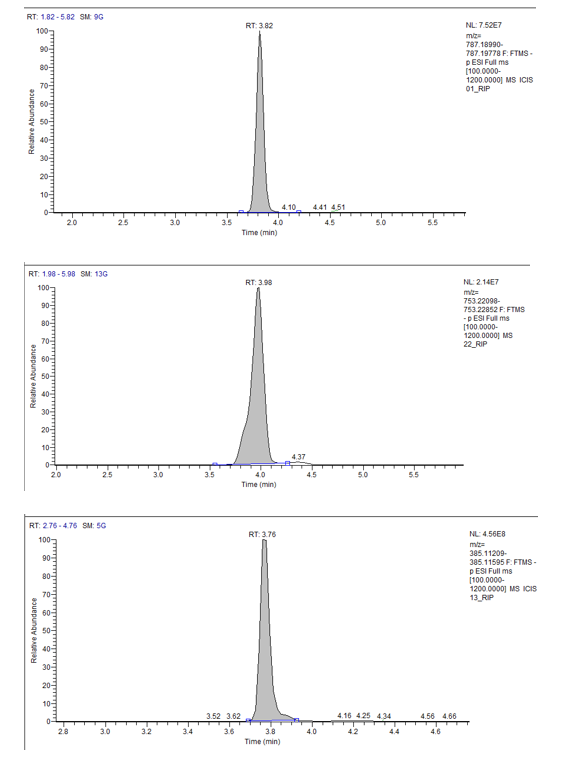

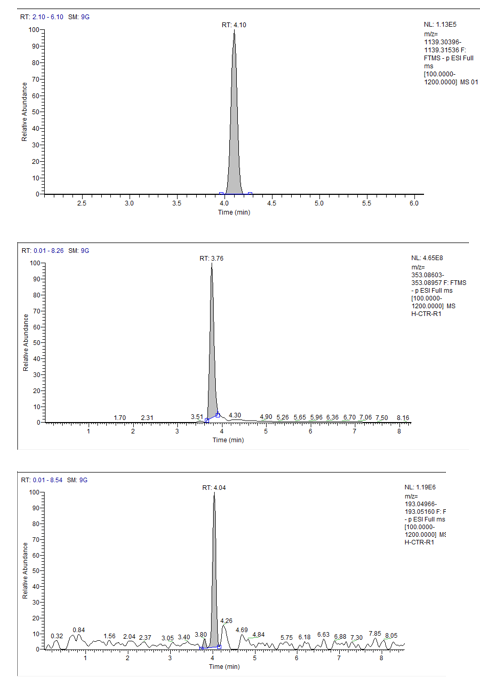


5-FQA

hydroxybenzoic acid

diosmin

myricetin

**Supplementary Figure 4** UHPLC-HRMS chromatogram of polyphenolic extract in freeze dried cardoon cells (for peaks identification see Supplementary Table 5).

**Supplementary Table 5** Mass specifications of the phenolic compounds analyzed in this study.

| Compounds | tr (min) | molecular formula | theoretical mass [M-H]^-^ | experimental mass [M-H]- | accuracy (Δ ppm) |
| --- | --- | --- | --- | --- | --- |
| 1,5-DiCQA | 4.03 | C25H23O12 | 515.11950 | 515.11957 | 0.14 |
| 3,4-DiCQA | 3.55 | C25H23O12 | 515.11950 | 515.11969 | 0.37 |
| 5-FQA | 4.10 | C17H19O9 | 367.10345 | 367.10315 | -0.82 |
| 3-FQA | 3.79 | C17H19O9 | 367.10345 | 367.10335 | -0.27 |
| 3-CQA | 3.91 | C16H17O9 | 353.08798 | 353.08783 | -0.42 |
| Coumaric acid | 3.88 | C9H8O3 | 163.03917 | 163.03912 | -0.31 |
| Quercetin-glucoside | 3.96 | C21H20O12 | 463.08835 | 463.08838 | 0.06 |
| Myricetin | 3.74 | C15H10O8 | 317.03009 | 317.03027 | 0.57 |
| Diosmin | 3.76 | C28H32O15 | 607.16699 | 607.16724 | 0.41 |
| Luteolin-glucoside | 3.89 | C21H20O11 | 447.09328 | 447.09283 | -1.01 |
| hydroxy-benzoic acid | 4.04 | C7H6O3 | 137.02442 | 137.02365 | -5.62 |
| Luteolin-rutinoside | 3.94 | C27H30O15 | 593.15119 | 593.15076 | -0.72 |
| Ferulic acid | 4.19 | C10H10O4 | 193.05063 | 193.05009 | -2.80 |

**Supplementary Figure 5 A-B** Growth changes of CcSADOE and CcFAD2.2KO lines for 28 days subculturing on solid Gamborg B5 culture medium. Fresh weight determination every seven days (A) CcSADOE lines. (B) CcFAD2.2KO lines. Data are presented as mean ± SD (n=24).

3
